# IGLoo enables comprehensive analysis and assembly of immunoglobulin heavy-chain loci in lymphoblastoid cell lines using PacBio high-fidelity reads

**DOI:** 10.1016/j.crmeth.2025.101033

**Published:** 2025-05-01

**Authors:** Mao-Jan Lin, Ben Langmead, Yana Safonova

**Affiliations:** 1 Department of Computer Science, Johns Hopkins University, Baltimore, MD 21218, USA; 2 Computer Science and Engineering Department, Pennsylvania State University, University Park, PA 16802, USA; 3 Huck Institutes of Life Sciences, Pennsylvania State University, University Park, PA 16802, USA

**Keywords:** lymphoblastoid cell line, LCL, immunoglobulin gene loci, IG, immunoglobulin heavy chain, IGH, assembly

## Abstract

High-quality human genome assemblies derived from lymphoblastoid cell lines (LCLs) provide reference genomes and pangenomes for genomics studies. However, LCLs pose technical challenges for profiling immunoglobulin (IG) genes, as their IG loci contain a mixture of germline and somatically recombined haplotypes, making genotyping and assembly difficult with widely used frameworks. To address this, we introduce IGLoo, a software tool that analyzes sequence data and assemblies derived from LCLs, characterizing somatic V(D)J recombination events and identifying breakpoints and missing IG genes in the assemblies. Furthermore, IGLoo implements a reassembly framework to improve germline assembly quality by integrating information on somatic events and population structural variations in IG loci. Applying IGLoo to the assemblies from the Human Pangenome Reference Consortium, we gained valuable insights into the mechanisms, gene usage, and patterns of V(D)J recombination and the causes of assembly artifacts in the IG heavy-chain (IGH) locus, and we improved the representation of IGH assemblies.

## Highlights

• IGLoo reports recombination events and measures cell line clonality from LCL datasets
• Non-canonical V(D)J recombination events are found in LCL datasets
• IGLoo detects artifacts in LCL-based IGH locus assemblies
• IGLoo reassembles the IGH locus and recovers missing IGH genes

## Motivation

While lymphoblastoid cell lines (LCLs) are widely used in genomics studies, few studies have evaluated the impact of somatic recombination on the immunoglobulin (IG) loci in these cell lines, despite their critical role in immunology research. To address this gap, we developed IGLoo, a toolkit designed to analyze recombination evidence in both read data and assemblies of the IGH locus. IGLoo provides deeper insights into how V(D)J recombination affects LCL-based datasets, highlighting that these cell lines do not perfectly represent the germline sequence.

## Graphical abstract

**Figure:**
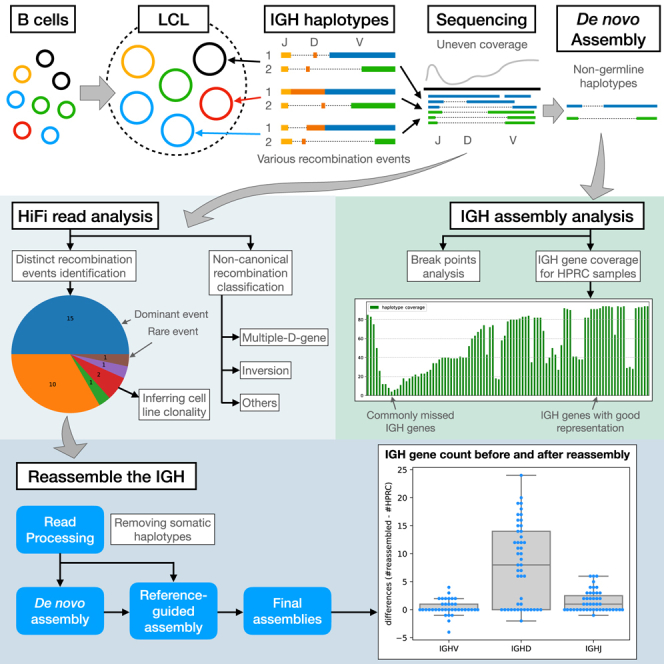

## Teaser

Lin et al. developed a software tool that detects both canonical and non-canonical V(D)J recombination events in LCL sequence datasets. They analyze artifacts in the IGH region from LCL-derived assemblies and improve germline representation by reassembling the IGH region and recovering missing IGH genes.

## Introduction

Immunoglobulin (IG) gene loci are essential for the development of B cell receptors (BCRs) and antibodies (Abs), which are one of two main components of the adaptive immune system. Most mammalian genomes contain three IG loci: one IG heavy-chain (IGH) locus and two IG light-chain loci, kappa chain (IGK) and lambda chain (IGL). The IGH locus comprises a series of variable (V), diversity (D), joining (J), and constant (C) genes, while the IGK and IGL loci have a similar structure but without the D genes. During B cell maturation, a process known as V(D)J recombination occurs, where one V, D (in case of IGH locus), and J gene segment are randomly selected and joined to form a rearranged V(D)J segment. In the IGH, D-J recombination precedes V-D recombination, resulting in the excision of the sequence between the selected D and J genes, followed by the excision of the sequence between the selected V gene and the newly formed D-J complex.^1^^,^^2^ These rearrangements, processed through double-strand breaks and subsequent repair at the recombination signal sequences (RSSs) flanking the selected IG genes, generate a diverse antibody repertoire and mount the potential to bind and neutralize a wide range of antigens. The genotype of these loci shape an individual’s antibody response, highlighting the importance of the germline diversity of IG genes and loci in adaptive immunity.^3^^,^^4^^,^^5^

Lymphoblastoid cell lines (LCLs) are a widely used system for providing a continuous source of human cells. LCLs are easy to prepare and maintain, and they have a low somatic mutation rate in continuous culture.^6^ LCLs can provide an ongoing source of DNA that is almost identical to original normal cells when compared using whole-genome sequencing (WGS).^7^ LCLs were used in several major consortium projects that surveyed human genetic variation, such as the 1000 Genomes Project,^8^ HapMap,^9^ Genome in a Bottle,^10^ and the Human Pangenome Reference Consortium (HPRC).^11^

On the other hand, previous studies also showed that the DNA derived from LCLs contains V(D)J recombinations and somatic hypermutation (SHM) events^12^^,^^13^ affecting IG loci and making them harder to genotype and assemble. This is because LCLs are generated by transforming B cells using Epstein-Barr virus (EBV). The original B cells likely already harbored V(D)J recombination events, as normal B cells typically do.

There have been a few attempts to profile human IG loci. Rodriguez et al.^14^ and Gibson et al.^15^ used PacBio single-molecule high-fidelity (HiFi) sequencing to target and study IG loci from LCLs. The former focused on the IGH locus, while the latter examined the IGL locus. Both papers report the germline genotypes of the IG loci and identify regions within the loci where information has been lost due to V(D)J recombination. A more recent study by Rodriguez et al.^3^ circumvented these challenges by using sequence data from peripheral blood mononuclear cells (PBMCs) or polymorphonuclear leukocytes instead of LCLs, thus avoiding the obstacles posed by V(D)J recombination. Nonetheless, LCLs are still widely used and easily accessible in existing databases. As a result, there remains a significant need for an accurate tool for IG profiling in LCL data.

HPRC aims to build a human pangenome reference to represent global genetic diversity. In their year-1 release, HPRC provided 47 phased, diploid assemblies from 15 subpopulations. All 47 samples are derived from LCLs. The samples were sequenced with PacBio HiFi sequencing with an average read depth of 39.7×, except HG002, which had average coverage >130×. The WGS Illumina short reads of the individuals’ parents are also available to phase the individual’s assembly. The HiFi reads of the individual and Illumina reads of the parents were assembled into phased assemblies with the trio-binning mode of Hifiasm.^16^ Although the assemblies are carefully curated, no particular measures have been employed to study the assembly quality in the IG loci and how they may have been affected by the LCL input. At this time, little is known about how representative the assemblies are of the germline IG sequence.

We present IGLoo, a toolkit for assessment and improvement of the IGH locus representation in LCL sequencing data. IGLoo profiles the somatic V(D)J recombination events in an LCL genome and measures their clonality. Then it improves the germline assembly of the IGH locus by removing the reads representing somatic haplotypes driven by V(D)J recombination and reassembling the dataset using Hifiasm and MaSuRCA.^17^ Finally, IGLoo assesses the quality of IGH locus assembly by analyzing the breakpoints, missing IGH gene segments, and the switching error of the assembly. IGLoo can also validate the newly assembled IGH gene segments with an independent Illumina short-read dataset.

We applied IGLoo to WGS LCL datasets collected from 47 individuals in the HPRC year-1 release. We observed diverse levels of clonality as well as varying quality of the assemblies in the IGH locus. By analyzing the HiFi reads, we also found evidence of a range of non-canonical V(D)J recombination events, which involve V, D, and J gene segments but do not align with either complete V(D)J or typical D-J recombinations. To our knowledge, IGLoo is the first work to systematically analyze and report non-canonical recombination events in long-read datasets and assemblies. We further applied the IGLoo reassembly framework to improve the assembly result of IGH locus. On average, IGLoo reassembled IGH locus covered 10 more IGH gene segments per individual than HPRC year-1 assemblies.

## Results

**Figure 1:**
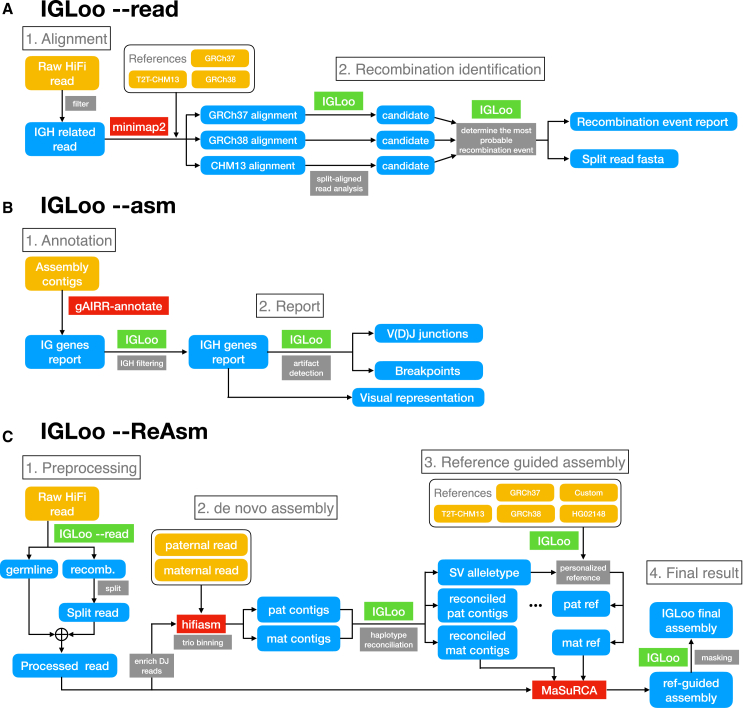
Pipeline diagram for all IGLoo modules (A) IGLoo --read pipeline. HiFi reads are aligned to three references: GRCh37, GRCh38, and T2T-CHM13. Split alignments are analyzed, and the most probable alignment out of the three references is selected to determine recombination events. (B) IGLoo --asm pipeline. The assembly is annotated with gAIRR-suite,^25^ and only contigs representing the IGH locus are analyzed. Assembly artifacts and IGH gene coverage are then reported. (C) IGLoo --ReAsm assembly pipeline. Somatic haplotypes are removed from the read data, and *de novo* assembly is performed with Hifiasm.^16^ The resulting contigs serve as the backbone for reference-guided assembly, with regions unsupported by read alignment masked in the final output. Custom: the custom IGH reference genome from Rodriguez et al.^3^ HG02148: *de novo*-assembled paternal haplotype of HG02148.

The IGLoo tool, which analyzes the IGH locus of human LCLs sequenced using PacBio HiFi reads, consists of three modules, as illustrated in Figure 1 and described in detail in STAR Methods. The first module, IGLoo --read, identifies and quantifies V(D)J recombination events present in a sample’s read alignment, offering insights into the recombination event frequency and IGH gene usage. The second module, IGLoo --asm, evaluates an assembly by cataloging present or missing IGH gene segments and pinpointing regions where the assembly was fragmented due to breakpoints. Lastly, the IGLoo --ReAsm module utilizes the output from IGLoo --read to modify input reads and construct refined *de novo* and reference-based assemblies, resulting in an improved assembly that more comprehensively captures the germline genome.

We applied the IGLoo modules to human LCL samples representing 47 individuals available at the HPRC.^11^ Each sample consists of WGS HiFi reads, phased genome assemblies, and parental Illumina read data.

### IGLoo --read profiles the V(D)J recombination events in LCLs

To identify the V(D)J recombination events in the WGS data, it is essential to consider the mechanism of the V(D)J recombination. V(D)J recombination initiates and terminates at RSSs flanking the selected V, D, and J genes. In IGH, a read carrying a V(D)J recombination event usually represents a deletion from one specific RSS to another RSS. WGS HiFi sequencing data are particularly advantageous for this task because typical V(D)J recombination junctions, including the connected V, D, and J segments, span less than 500 bp, whereas the entire non-canonical recombination structure can exceed thousands of base pairs (results section: Non-canonical V(D)J recombination events). HiFi reads are long enough to cover these events while also providing sequence context on either side of the recombination. Moreover, WGS HiFi enables detection of unusually long recombination events, such as RSS skipping^18^ and the LAIR-1 insertion^19^ as well as discovery novel types of non-canonical recombination events.

To profile the V(D)J recombination events in a sample, the IGLoo --read module takes either read alignments (BAM/CRAM) or HiFi reads (FASTA) and collects the reads spanning recombination events. When a HiFi read overlaps a V(D)J recombination event, a read aligner like minimap2^20^ will generally yield an alignment with distinct segments of the read aligning to either side of the recombination event (Figure S1). We call this a split alignment, and we say the read is a split-aligned read. Since the IGH locus is highly polymorphic,^14^ and no standard human reference genome covered all IGH gene segments, IGLoo --read utilizes a multi-reference strategy (STAR Methods: Profiling V(D)J recombination events using HiFi reads) to maximize the number of HiFi reads showing evidence of V(D)J recombination. IGLoo --read then reports the best-supported recombination events based on alignments to three human reference genomes: GRCh37,^21^ GRCh38,^22^ and T2T-CHM13.^23^ The profile of the recombination events is then used to compute the gene usage and clonality of the cell line.

IGLoo --read scans and analyzes only the reads fully or partially mapped to the region stretching from J gene to D gene. In this way, we can detect reads that are split aligned due to V-D and D-J recombination or a combination of both. That is, we expect any recombination event involving a V gene to also involve a J or D gene. As a result, we can detect them simply by analyzing the split alignments in the region inspected. This way, reads that are split-aligned because of structural variations (SV) in the V gene locus will likely not be included in the analysis.

Since V(D)J recombination initiates and terminates at the RSSs of the selected V/D/J genes, when a read is split aligned to two IGH gene segments due to such an event, we anticipate the split sites to be in close proximity to the RSSs. Alongside recombination-driven deletions, non-genomic nucleotides contributing to the diversity at the IG gene junctions and somatic hypermutations (SHMs) can also occur on the recombined fragment. However, HiFi reads are resilient to mismatches and small indels caused by SHMs and junctional diversity, allowing for reliable identification of split sites. We assessed the distance between split sites and the nearest V, D, J RSS for all split-aligned reads. Of the 1,730 split-aligned reads across the 47 samples, totaling 3,357 split segments with some alignments split into more than two segments, over 93% of segments were within 50 bp of an RSS (Figure S2). Therefore, we considered split-aligned pairs where the split position is within 50 bp of the RSS as “confident” events and focused our gene usage analysis solely on these confident events.

#### Commonly used genes across cell lines

**Figure 2:**
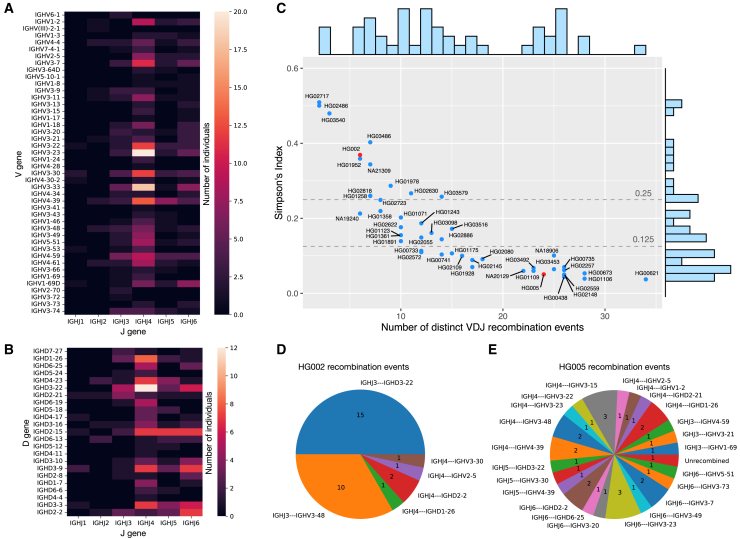
Heatmap and clonality analysis of V(D)J recombination across HPRC samples (A) Heatmap showing number of individuals (out of 47 HPRC samples) with a specific combination of V and J genes in complete V(D)J recombination. Each cell represents a V-J pair, with the color indication the number of individuals carrying this recombination. (B) Heatmap showing number of individuals with a specific combination of J and D genes in D-J recombinations. (C) Number of unique recombination event in a sample versus the SI of the reads supporting each clone. The positions of HG002 and HG005 are highlighted in red. Dashed lines indicate the thresholds for monoclonal and polyclonal cell lines, set at 0.25 and 0.125, respectively. (D and E) (D) Unique recombination events and number of reads supporting the event for cell line HG002 and (E) cell line HG005.

To detect the commonly used IGH genes across the cell line, we analyzed the HiFi reads that showed evidence of V(D)J recombination events and collected the respective V, D, or J genes used in these events. In Figure 2A, we show the number of individuals having confident canonical V(D)J recombination events with specific J and V gene pair and in Figure 2B the number of individuals having confident D-J-only recombination, i.e., partial recombination, with specific J and D gene out of the 47 samples. The most widely used J gene across the samples is *IGHJ4* in both complete V(D)J and partial D-J recombination events, the same as the result in Rodriguez et al.^3^ In D-J recombination events, the most widely used D gene was *IGHD3-22*, which is consistent with the analysis of expressed V(D)J recombinations (Rep-Seq) described in Safonova and Pevzner.^18^ For complete V(D)J recombination events, the most widely used genes were *IGHV3-23* and *IGHV3-33*, which is very similar to the gene usage analysis obtained using short-read (Illumina) repertoire sequencing for both isotype immunoglobulin M (IgM) and immunoglobulin G (IgG) described by Rodriguez et al.^3^

Our analysis also revealed gene usage patterns that are challenging to detect using Rep-Seq data. The first involves an 11-nt-long D gene *IGHD7-27* that is difficult to distinguish from random matches in Rep-Seq data. In the LCL data, the D gene *IGHD7-27* was detected and found to participate in partial V(D)J recombination events with *IGHJ6* and *IGHJ3*. In total, we identified all six J genes, 23 D genes, and 44 V genes being used in the recombination events. Four out of the 44 V genes, *IGHV3-22*, *IGHV(III)-2-1*, *IGHV1-17*, and *IGHV3-41* are classified as pseudogenes in the IMGT database.^24^ *IGHV3-22* were used in 12 samples, while the remaining three V genes were only used in single samples (Figure S3).

#### Clonality of the cell lines

We collected all the V(D)J recombination events of each sample and used Simpson’s index (SI) to measure the clonality of the sample. This is calculated as

(Equation 1) $$\text{SI}=\sum_{n\;\text{in}\;\text{events}}n^{2}/N^{2}$$

where *n* is the number of reads in a unique V(D)J recombination event, and *N* is the total number of reads containing V(D)J recombination event. The higher the SI, the more monoclonal the sample is.

We plot all the 47 samples according to their number of distinct V(D)J recombination events and SI in Figure 2C. The negative trend is expected, since SI will tend to decrease with increasing number of distinct events. The Spearman’s rank correlation coefficient *r* between SI and the number of distinct events is −0.9387 in this case, indicating a strong negative correlation. For a monoclonal sample, we expect SI to be near 0.5, representing one dominant recombination event per haplotype. This is the case for a few samples that appear toward the left of Figure 2C. Most samples, though, have SI less than 0.5, indicating a degree of polyclonality. There was no clear separation between the SI values for monoclonal and polyclonal samples; rather, they formed a continuous spectrum. Note that our method will generally underestimate polyclonality due to the finite depth of WGS HiFi samples. For instance, the average coverage in these samples was about 38× in the IGH locus, impairing our ability to measure SI below about 0.03.

To illustrate the differences between monoclonal and polyclonal samples, we picked one sample (HG002) situated close to the monoclonal end and one sample (HG005) situated close to the polyclonal end for comparison. The positions of the two samples are highlighted in red in Figure 2C, and their V(D)J recombination events are shown in Figures 2D and 2E, respectively. The majority of the events for HG002 fall into two categories, consistent with this being a cell line with two haplotypes, each with a dominant recombination event. For HG005, on the other hand, the 33 reads showing recombination evidence were categorized into 23 distinct events, with no single event carrying more than three reads.

While there is no definitive boundary between monoclonal and polyclonal samples, for simplicity we defined samples with SI greater than 0.25 (half of the upper limit of 0.5) as monoclonal and samples with SI less than 0.125 (half of the previous threshold) as polyclonal. Using these thresholds, 13 and 22 samples were classified as monoclonal and polyclonal, respectively, and 12 samples with SI values above 0.125 and below 0.25 were classified as in between.

A previous study^13^ proposed an alternative method for calculating clonality, defined as the fraction of sequencing reads attributed to the dominant event. However, this approach is not directly applicable to our analysis, as Rodriguez et al.^13^ consider only complete V(D)J recombination, whereas we also account for D-J only and non-canonical recombination events. Our definition of monoclonal cells roughly aligns with the >50% category described in Rodriguez et al.^13^ (Figure S4).

#### Non-canonical V(D)J recombination events

A total of 709 distinct events were identified in the HPRC samples. Each distinct event represents a unique V(D)J recombination event or the same event observed in different individuals. Among these events, 13 (2%) are unrecombined events, 466 (66%) are canonical complete V(D)J recombination events, and 191 (27%) are canonical D-J only recombination events. The remaining 35 (5%) events are non-canonical; we classified 23 as multiple-D-gene recombination and 12 as inversion events.

**Figure 3:**
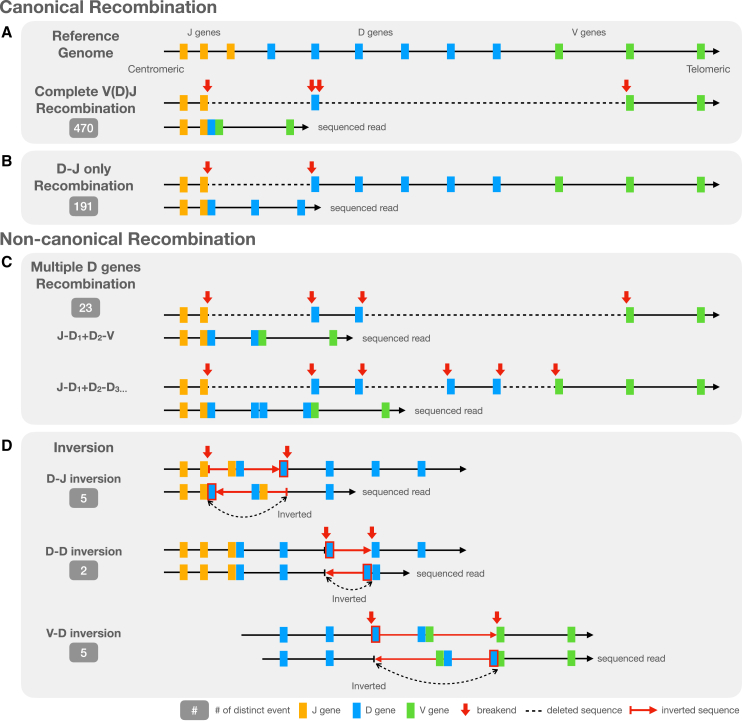
Canonical and non-canonical recombination events (A–D) Illustration of canonical (A and B) and non-canonical (C and D) recombination events. (A) A complete V(D)J recombination event relative to the reference genome. (B) Partial (D-J only) recombination event. (C) Non-canonical multiple-D-gene recombination events. (D) Non-canonical events involving an inversion between a D-J pair, D-D pair, or V-D pair.

In a complete V(D)J recombination event, both the D-J recombination and V-D recombination utilize the same D gene. That is, the RSSs on both sides of one specific D gene are connected to other specific J and V genes via split alignments (Figure 3A). A partial recombination event occurs when the recombination process is halted after the sequence between J and D genes is deleted. However, the sequence upstream of the selected D gene is intact and remains in its germline form (Figure 3B).

We observed two types of non-canonical recombination events. The first type is multiple-D-gene recombination, which involves at least one additional D gene recombination (Figure 3C). The second type involves an inversion additional to V-D or D-J recombination (Figure 3D). Events like these often include non-coding regions of the IGH locus and are unlikely to produce a functional end product. However, they can be confused with SVs in the germline genome and thus need to be discarded during downstream analyses.

Figures 3B–3D illustrates the observed types of the incomplete and non-canonical recombination events, which we also detail here.

##### Partial recombination (D-J only or V-D only)

D-J-only recombinations are the second most prevalent recombination event, surpassed only by complete recombination events. They are observed in 44 out of 47 samples. The distinction between partial recombination event and complete recombination is evident due to the existence of the germline flanking sequence on the $5^{\prime }$ end of the chosen D gene. The lengths of human D genes do not exceed 40 bp, making them easy to span by HiFi reads.

Nearly all observed partial recombination events are D-J-only recombination exclusively. However, an exception was noted in the sample HG03492 (Figure S5), where V-D-only recombination occurred independently. In this case, the sequence on the $3^{\prime }$ end of the D gene split sites extend to the region between IGHJ and IGHD loci, indicating the absence of the D-J recombination in the event.

##### Multiple-D gene

The most common type of non-canonical recombination events involves more than one D gene and was detected in 20 out of 47 samples in HPRC.

In this scenario, V(D)J recombination uses the $5^{\prime }$ RSS of one D gene and the $3^{\prime }$ RSS of another D gene located downstream of the first one, thus creating a virtual “ultralong” (and not necessarily productive) D gene. In Safonova and Pevzner,^18^ this scenario was referred to as RSS skipping. Two examples of RSS skipping are illustrated on an IGV screenshot that shows alignments of HiFi reads from HG02257 (Figure S6).

Figures S6A–S6C illustrate a recombination event where the HiFi read is split aligned into three segments, with two long deletions between *IGHJ2* and *IGHD4-23*, and between *IGHD6-19* and *IGHV3-49*. The panels Figures S6D–S6F provide details of the HiFi read, and the full recombination event utilizes the $3^{\prime }$ RSS of *IGHD4-23* and the $5^{\prime }$ RSS of *IGHD6-19*.

Figure S6G presents another recombination event in the same sample, where the HiFi read is also split aligned into three segments, with two long deletions between *IGHJ4* and *IGHD5-18*, and between *IGHD4-17* and *IGHD2-2*. Figures S6H and S6Ishow the details of the HiFi read. In summary, the simplest form of multiple-D-gene recombination event occurs when D-J and V-D recombination involve different D genes, whereas more complex multiple-D-gene recombination can involve additional D-D recombination along with D-J recombination.

##### Inversion

Figure 3D illustrates how an inversion can occur in addition to partial or multiple-D-gene recombination. The inversion is initiated and terminated at the RSSs of two IGH gene segments. In all observed cases, at least one D gene was involved in the inversion. This type of non-canonical event is less common than multiple-D-gene events, with only five, two, and five unique events involving D-J, D-D, and V-D inversion events, respectively.

In instances where the D-J gene pair is inverted, the sequence between the RSS of the selected J gene and the $5^{\prime }$ RSS of the selected D gene is inverted. An example of D-J inversion from individual HG02622 is shown in Figures S7A–S7E.

In the case of a D-D gene pair inversion, the sequence between the $3^{\prime }$ RSSs of two consecutive D genes is inverted. Similarly to the D-J inversion, the two D genes are connected together at the $5^{\prime }$ end of the inversion in the read sequence. An example of a D-D inversion from individual HG01978 is shown in Figures S7F–S7J.

In the case of a V-D gene pair inversion, the sequence between the selected V RSS and the $3^{\prime }$ RSS of the selected D gene is inverted. Due to the large size of the V-D inversion structure, no single read has been observed to cover the entire structure. However, HiFi reads can still span one end of the inversion. As shown in Figure 3D, the left-breakend of the inversion results from the connection of the two RSSs of the selected D and V genes, while the right-breakend represents an inverted connection between the selected V and D genes. An example of a HiFi read covering the inversion left-breakend of a V-D inversion from individual HG00621 is shown in Figures S8A–S8F.

An additional example involving an inverted V sequence from individual HG00741 is shown in Figures S8G–S8J. As depicted in Figure 3D, if the V-D recombination in the original sequence occurs close to the D gene selected for the inversion, a read may cover a structure containing two junctions between two sets of V-D pairs. Note that, in a standard V-D recombination, the V and D genes share the same orientation, whereas, at the inversion breakpoint, the orientations of the two genes are reversed. Figures S8G–S8J illustrates a case in which two V-D junctions are covered.

Most of the observed inversion events can be explained by a D-J recombination combined with a D-J or D-D inversion, or a V-D recombination combined with a V-D inversion. Due to the limited read length, it is unclear whether both D-J recombination and V-D recombination occur simultaneously in the cases we observed. However, a complex non-canonical recombination event was identified from the individual HG02559. The alignment and detailed read sequence are shown in Figure S9, where the non-canonical recombination event consists of a D-J recombination, a D-J inversion, and an additional translocation.

Comparing to complete V(D)J recombination and D-J only recombination events, non-canonical recombination accounts for only 5% of the distinct events. However, they are still present in 25 out of 47 samples. These events include long-range rearrangements such as deletions and inversions between RSSs of the V, D, and J genes and are arranged in ways different from canonical V(D)J recombination. Understanding the mechanisms behind these events is essential for differentiating reads carrying somatic events from reads carrying germline SVs in the IGH locus.

### IGLoo --asm measures the somatic effect on the HPRC assemblies

For LCL datasets, V(D)J recombination impedes the assembler’s ability to accurately assemble the germline genome sequence. For example, recombination depletes read coverage in the germline sequence between the V and J genes; this depletion effect is particularly severe in D and J gene loci.^13^ Polyclonality leads to the emergence of reads originating from somatically rearranged haplotypes, which might violate the assembler’s expectation of a strictly diploid and nearly uniformly covered genome. Consistent with this, we observed that the HPRC assemblies were not contiguous in the IGH locus and that IGH gene segments were lost during assembly.

The IGLoo --asm module is designed to profile the IGH locus in genome assemblies, locate V(D)J junctions in the contigs and breakpoints in the assembly with respect to the germline IGH gene segments, and identify IGH gene segments missing from the assembly. IGLoo --asm first calls gAIRR-suite^25^ to annotate the IGH gene segments on the assembly. It identifies all contigs overlapping IGH gene segments and filters out the contigs containing only IGH gene segments with more than 15 mismatches comparing to documented IGH genes from IMGT. These are likely to contain “orphon” genes, which have homology to IGH genes but are located outside the main IGH locus (STAR Methods: Analyzing the personal assemblies). Finally, IGLoo --asm compares the filtered contigs to the germline IGH locus to situate V(D)J junctions and breakpoints with respect to the IGH gene segments.

**Figure 4:**
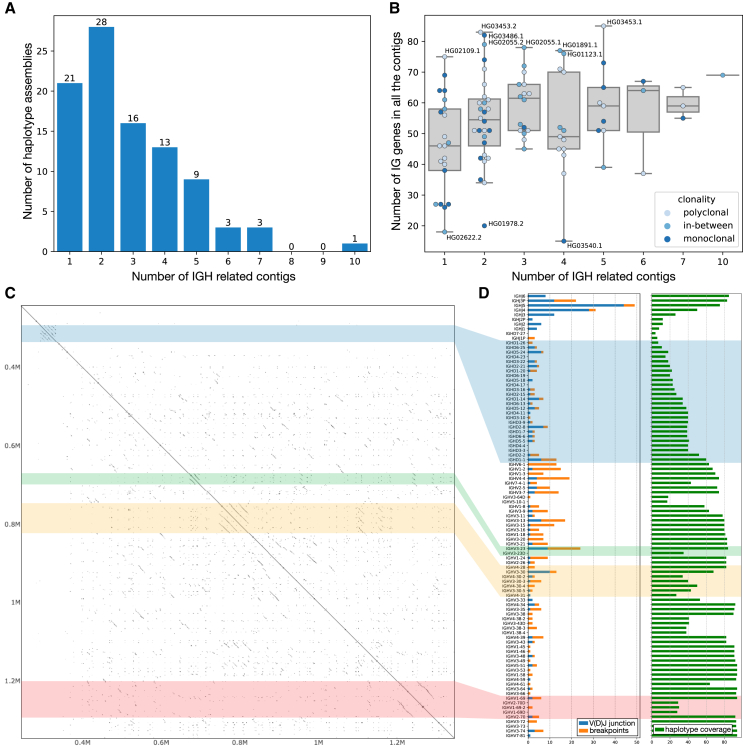
Assembly metrics and across HPRC samples (A) The accumulated bar plot of the number of contigs in one haplotype assembly of HPRC. (B) The number of functional IGH genes covered by all the contigs in each haplotype assembly; note genes on duplicated sequences are counted only once. Dots represent haplotype assemblies, and the hues of the dots represent the clonality of the cell line according to SI and our definitions of monoclonal and polyclonal samples in results section Clonality of the cell lines. (C) Self-aligned dot plot of the sample NA19240 haplotype 1 assembled by IGLoo with ModDotPlot.^27^ (D) (Left) The accumulated V(D)J junctions and breakpoints of the 94 HPRC haplotypes. How the IGH gene segments are related to the V(D)J junctions and breakpoints, including the disjoint, overlapping, and duplication cases, is depicted in Figure S10. (Right) The occurrence of each IGH gene segment in the assemblies of the 94 haplotypes, counted as 1 per haplotype if covered. The colored strips across (C) and (D) show the relative position of the repetitive regions in the dot plot and bar plot.

We used IGLoo --asm to profile all of the genome assemblies from the HPRC project. Figure 4A shows the number of contigs for the 94 HPRC haplotype assemblies (two assemblies per individual). Twenty-one (22%) of the haplotypes have a single contig covering all or part of the IGH locus. Twenty-eight (30%) of the haplotypes have the IGH locus split into two contigs, and 45 (48%) haplotypes have the locus split into three or more contigs. In cases where the IGH locus was covered by a single contig, the assembly sometimes suffered from missing genes due to V(D)J recombination events or other assembly issues. Figure 4B shows the total number of V genes (without pseudogenes), D genes, and J genes covered by all the contigs for each haplotype. Note that some assemblies have duplicated sequences, and we counted the same IGH gene only once in these cases. According to IMGT,^24^ there are 95 such genes in the IGH locus, but the number varies due to germline variation. Since the 95 genes account for most insertions in the IGH locus, haplotypes are more likely to have fewer genes than to gain additional ones.

A joint analysis of sample clonality and assembly quality, defined in this study as a combination of the number of IGH genes covered and the contiguity of the haplotype assembly, did not reveal clear relations between these two factors (Figure 4B). For example, some monoclonal cell lines produced contiguous assemblies covering a high number of IGH genes, such as HG03486 haplotype 1 (paternal). On the other hand, HG03540 haplotype 1 (paternal), despite being monoclonal, resulted in a fragmented assembly comprising four contigs and covering fewer than 20 IGH genes. Similarly, the assembly qualities of polyclonal cells also exhibit considerable variability.

Another challenge to correct assembly of the IGH locus is the presence of repeats. We focused on four common repetitive regions in the part of IGH locus spanning V, D, J genes illustrated in a self-dot plot for NA19240’s paternal haplotype (Figure 4C). The dot plot showed the repetitive regions in the self-alignment of the complete NA19240 paternal IGH locus we reassembled in the results section: IGLoo --ReAsm can improve the IGH assemblies. The four regions with parallel diagonal lines are the common repetitive regions on the IGH locus: the IGHD gene locus (the blue block), the duplication of the *IGHV3-23* (the green block), a nearly tandem repeat containing *IGHV3-30*-like and *IGHV4-30*-like genes (the yellow block), and the locus containing copies of IGHV1-69 genes (the red block). Previous studies show that these loci represent hotspots for SVs,^3^ and some haplotypes in the HPRC population are also characterized by different number of copies of *IGHV3-23*, *IGHV1-69*, or *IGHV3-30*-like genes.

There are two main artifacts in the assemblies: the first is breakpoints, which result in contig fragmentation and potential gene loss. Breakpoints occur at the ends of contigs that do not span the entire IGH locus, leaving the contig ends within the locus. Two adjacent contigs can be disjoint, overlap each other, or one can be contained in the other (as shown in Figure S10A). Note that only disjoint breakpoints contribute to the loss of IGH gene segments. In all these cases, the breakpoints are defined as the terminal positions of the contigs.

Another type of assembly artifact is the V(D)J junction, where the assembly shows evidence of somatic recombination instead of germline haplotype. We detected these V(D)J junctions by searching for contigs that connect V to J, V to D, or D to J genes (Figure S10B). Although the V(D)J junctions technically do not affect assembly continuity, they can lead to omission of genes from the assembly. For each breakpoint and V(D)J junction in the 94 haplotype assemblies, we marked the nearest J, D, or non-pseudo V genes and summarized the result in the left bar chart of Figure 4D. The colored strips indicate which repeat regions in the dot plot (Figure 4C) correspond to these bars. Since all the D genes except *IGHD7-27* are included in the D gene repeat block, the blue transparent block roughly divided the J/D/V genes in the Figure 4D. All the genes below the block are the V genes, and the genes above the blue transparent block except *IGHD7-27* are J genes.

The right bar plot of Figure 4D shows the occurrence of each IGH gene segment in the 94 haplotype assemblies. Since some haplotype assemblies have duplicated contigs, each gene is counted only once per haplotype. In the region spanning *IGHJ6* to *IGHV4-4*, the haplotype coverage of the IGH gene segments initially declines and then increases, with a minimum at *IGHD7-27*, which aligns with the read depletion in the J and D gene loci. Additionally, the haplotype coverage also fluctuates in several V regions, starting from *IGHV3-64D*, *IGHV3-23D*, *IGHV4-30-2*, *IGHV4-38-2*, and *IGHV2-70D*. Three of these fluctuations are in consistent with the repetitive blocks. These fluctuations are likely the result of population-level SV, since there are common complex SV or deletions documented in these regions.^3^

The left table in Figure 4D shows that V(D)J junctions are enriched in the J genes, consistent with the smaller number of J genes and the fact that each V(D)J junction must include one J gene. Combined with the observation that most missing IGH gene segments are J and D gene segments, we conclude that V(D)J recombination and the associated drop in read coverage of germline sequences during recombination significantly contribute to assembly artifacts and the loss of IGH gene segments in the IGH assemblies.

### IGLoo --ReAsm can improve the IGH assemblies

Assemblies derived from LCLs can be fragmented or can contain other flaws. The IGLoo --ReAsm module improves the accuracy of the germline assembly obtained from LCLs. This module deploys two distinct reassembly methods, one that uses the Hifiasm *de novo* assembler^16^ to assemble the backbone of the IGH locus, and then the backbone is further improved by the reference-guided assembler MaSuRCA^17^ (Figure 1C).

Two main challenges of the LCL data are the somatic haplotypes and the low read depth around the J and D gene loci. The V(D)J recombination events in the dataset create non-germline haplotypes, which the assembler cannot automatically disregard in favor of the germline haplotype. Low depth in the region between J and D genes causes fragmentation and loss of IGH gene segments.

To tackle the somatic haplotype problem, IGLoo --ReAsm preprocesses the raw HiFi reads before reassembling them with Hifiasm. Based on the analysis of IGLoo --read, IGLoo --ReAsm collects all the reads showing evidence of V(D)J recombination breakpoint, indicated by split alignments on two sides of IG gene RSSs. These reads are then split at the breakpoint positions. To mitigate the effect of reference bias, IGLoo --ReAsm selects the best mapping result from the three reference genomes used by IGLoo --read (STAR Methods: Profiling V(D)J recombination events using HiFi reads). By doing the splitting, the somatic haplotypes are broken. On the contrary, the germline haplotypes remain intact in the read pool.

IGLoo --ReAsm also performs read enrichment to counter the read depletion in the region. Enrichment is achieved by creating artificial copies of reads mapping to the region between J and D genes, doubling the read depth in the region and giving the *de novo* assembler a stronger basis for assembling the region without fragmenting the contigs and losing D genes.

#### *De novo* assembly results

To produce an initial version of the improved germline assembly, IGLoo --ReAsm runs Hifiasm with the trio-binning method. Rather than use the full set of Illumina short reads from the individual’s parents, we extract and use only the parental reads mapping to the IGH locus. Since the parental data also originate from LCLs, it can suffer from the presence of somatic haplotypes and low read depth in the IGH locus, thus making the phasing information less reliable compared to other regions of the genome. Because somatic haplotypes are hard to identify using Illumina short reads, we do not attempt to remove reads containing V(D)J recombination events from the parental datasets. The assembled contigs are later used as the backbone for next stage, assembly and polishing.

**Figure 5:**
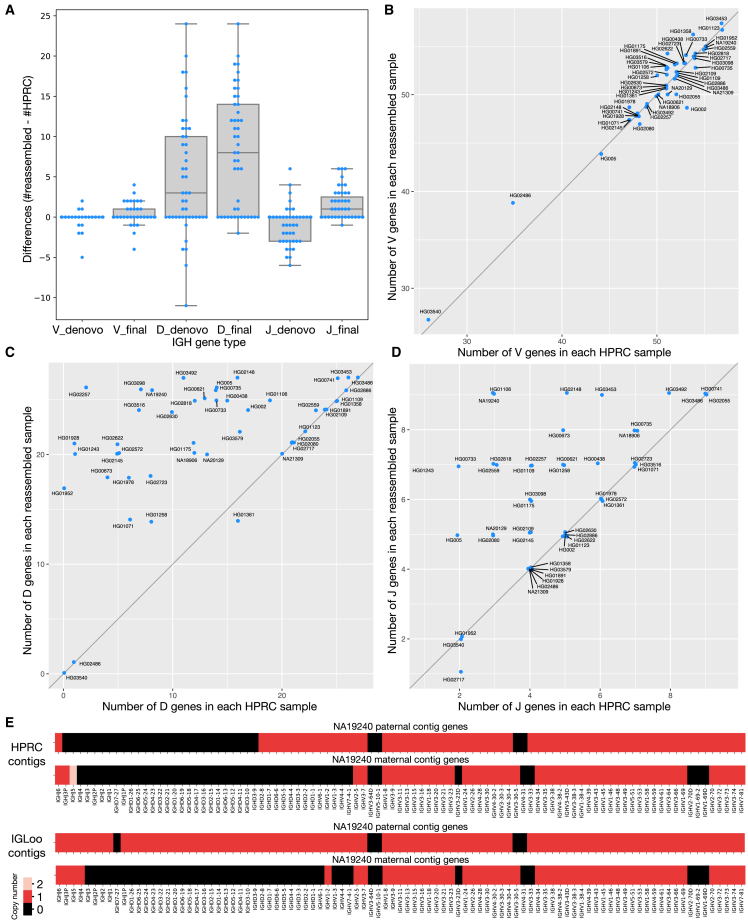
IGH gene-number differences between HPRC assemblies and IGLoo reassemblies (A) The distribution of gene-number differences between *de novo* assembly contigs and HPRC assemblies, and the differences between final assemblies and HPRC assemblies. _denovo, the numbers from *de novo* assemblies; _final, numbers from the final masked assemblies. Each dot represent one sample. Note that there are more dots crowded in the 0 entries, which exceed the width of the box. (B) The V gene-number differences of HPRC assemblies and reassembled IGH locus. (C) The D gene-number differences. (D) The J gene-number differences. (E) The gene number in its relative position on the sample NA19240 before and after IGLoo --ReAsm. All D and J genes are analyzed, but only functional or ORF V genes are analyzed for simplicity.

We use the IGLoo --asm to evaluate our reassembled genomes. Figure 5A shows the difference in the number of IGH gene segments between HPRC assemblies and the *de novo*-assembled contigs from processed reads. Boxes marked “_denovo” show the difference in count of the V, D, and J genes between HPRC and *de novo* assemblies on processed reads. For most individuals, the number of V genes stayed roughly equal, with the average number of differences being −0.15. The number of D genes increased, with the average change being +4.87 per individual. However, the number of J genes decreased by −0.94 on average.

The increase in the number of D genes can be explained by the preprocessing of the HiFi reads, which removes the somatic haplotype and increases the read depth in D gene locus. The decrease in the number of J genes is more complicated. Since most recombination events involve a J gene, raw reads covering J genes are likely to span a split site. As a result, preprocessed reads covering J genes are often clipped near a J gene. This clipping, combined with the possibly presence of SHMs and class switch recombination events occurring among the constant genes at the downstream sequence of the J genes, can cause fragmentation in the J gene locus. On the other hand, assembling from raw reads can generate some somatic haplotype in which a few J genes can still be preserved.

#### Reference-guided assembly

To recover the lost J genes in the *de novo* assembly stage, and to polish the assembly result, IGLoo --ReAsm uses the reference-guided assembler MaSuRCA.^17^ First, IGLoo --ReAsm uses the information in *de novo*-assembled contigs to generate a personalized reference genome for each of the individual’s haplotypes. The contigs, personalized reference, and processed reads are then fed to the MaSuRCA pipeline for scaffolding, gap filling, and polishing. Finally, IGLoo --ReAsm masks portions of the assembly that lack read support, since these portions come from the reference provided to MaSuRCA rather than from the input reads.

An SV allele is defined as a large haplotype that differs from the reference genome. IGLoo --ReAsm is able to detect the prevalent SV allele type from the *de novo*-assembled contigs. Sometimes, the allele type of a contig can contradict that of the other contig in the same paternal or maternal group. The contradiction may come from mis-categorizing of a contig from the other parental group due to the issue stated in the results section: De novo assembly results. IGLoo --ReAsm detected the SV haplotype and attempted to reconcile allele types by swapping conflicting contigs between paternal and maternal groups. Three samples—HG01891, HG03453, and NA18906—could not be reconciled through contig swapping. We addressed these three samples manually. After the reconciliation, a personalized reference is generated. IGLoo --ReAsm used the backbone of T2T-CHM13 and stitched different haplotypes of the SV according to the information provided by *de novo* contigs (STAR Methods: Personalized reference genome)

After the reference-guided assembly, IGLoo --ReAsm realigned the HiFi reads back to the MaSuRCA output and masked out regions without any support from the read alignments or *de novo*-assembled contigs. These are regions likely originating from the guide reference rather than from the input reads.

We again used IGLoo --asm to evaluate the final masked assemblies. Figures 5B–5D show the differences in V, D, and J gene counts between HPRC assemblies and the final masked IGLoo assemblies. Each dot represents an individual, and most dots are located on the diagonal line or in the upper left triangle, showing that IGLoo usually recovered an equal or greater number of IGH gene segments compared to the original HPRC assemblies. Figure 5A shows the gene-number differences from HPRC assemblies. The box marked with “_denovo” are the numbers of *de novo*-assembled contigs, and the numbers of the final assemblies are shown in the boxes marked with “_final.” In general, *de novo* assembly recovered some D genes while losing some J genes. On the other hand, the reference-guided method recovered the missing J genes from *de novo* assembly and also recovered more D genes. Note that, in Figure 5A, some of the 0-difference dots are not shown because they exceed the width of the box. Out of the 47 samples, 13 had an increased number of V genes and five had a decreased number. Thirty-three samples had an increase in D genes and one has a decreased number. Twenty-four had an increased number of J genes and one has a decreased number. The average V, D, and J gene differences were 0.32, 8.21, and 1.43 respectively.

Figure 5E illustrates one example NA19240 of HPRC assemblies alongside the final IGLoo masked assemblies. The genes on the contigs are arranged by the IGH locus. Both haplotypes of the HPRC assembles exhibited V(D)J recombination events, whereas the IGLoo --ReAsm assembly recovered the paternal haplotype into its unrecombined form. Note that the information of the D genes at the maternal haplotype is likely lost in the cell line (Figure S11). As a result, IGLoo --ReAsm barely recovered more IGH gene segments from the maternal haplotype.

#### Evaluation of the IGLoo reassembled genomes

We evaluated the phasing quality of the IGLoo assemblies using the yak trioeval tool,^16^ comparing them to the original IGH regions in the HPRC assemblies. Figure S12A illustrates the switching-error rate of each assembled haplotype before and after IGLoo reassembly. The average switching-error rate for the IGLoo reassemblies was 0.057, compared to the average of 0.071 of the original HPRC assemblies. IGLoo reassembly decreased the switching-error rate. Both the HPRC and IGLoo assemblies exhibited significantly higher switching-error rate in the IGH region than the overall genome assemblies, which reported an average switching-error rate of 0.0067 as noted by HPRC.^26^

The increased error rates in the IGH region likely result from the challenges posed by V(D)J recombination and SHMs, which complicate both the assembly and evaluation processes. Furthermore, the parental data used in the evaluation, derived from LCLs, may also be affected by these factors. Overall, the IGLoo reassembly process successfully recovered additional IGH gene segments in the sample while maintaining a switching-error rate comparable to that of the original assemblies.

To further validate the newly assembled IGH gene segments, we aligned Illumina short reads from 43 HPRC samples, which were not used in the reassembly process, to the IGLoo assemblies. A gene was defined as confident if it was fully covered by the reads with an edit distance of no more than 1 between the read and the assembled gene. Among the 600 newly assembled D gene segments, 493 were fully covered and 488 were classified as confident. Similarly, among the 102 newly assembled J gene segments, 51 were fully covered, with 46 classified as confident. Overall, 81% and 45% of the newly assembled D and J gene segments are confidently supported by short reads (Figures S12B and S12C).

#### Comparison with IGenotyper

IGenotyper^14^ is another method for profiling and assembling the IGH locus. We applied IGenotyper to the HPRC dataset in order to compare it to IGLoo. For efficiency, we used the same inputs as were provided to the IGLoo --ReAsm pipeline, where the reads have already been aligned (by HPRC) and filtered (by IGLoo) to the IG loci.

We ran IGenotyper on the 47 HPRC samples without conducting any manual curation as described in Rodriguez et al.,^14^ we used the output file igh_contigs.fasta from IGenotyper assembly step. We employed IGLoo --asm to evaluate the IGenotyper assemblies and compared them to the IGLoo --ReAsm results in the same manner as the comparison with HPRC assemblies. Figure S13A illustrates the differences in IGH gene numbers between the two methods. The distribution of the differences resembles the comparison between HPRC assemblies and IGLoo --ReAsm. Specifically, while the number of IGHV genes remains comparable, IGLoo --ReAsm exhibits a significant increase in IGHD and IGHJ genes. On average, the V gene difference is 0.83, D gene difference is 11.40, and the J gene difference is 3.68. Figure S13B shows the total IGH gene numbers in the samples between the two methods. All 47 samples either favor IGLoo --ReAsm or show no difference in the gene numbers. This is as expected, since IGLoo --ReAsm additionally removes somatic haplotypes before assembly.

## Discussion

We presented the IGLoo software tool, which implements several specialized methods for elucidating both the germline and somatic natures of the IGH locus when sequenced using HiFi reads from an LCL. IGLoo fills a gap in the current landscape of tools for *de novo* assembly of HiFi data, which treat data derived from LCLs as though they represented a pure germline sequence.

We used IGLoo to study the non-canonical V(D)J recombination events in the HPRC read data, finding that non-canonical events made up approximately 5% of all recombination events in the cell line. The length of HiFi reads facilitates analysis of somatic events involving IG gene segment inversions and multiple-D-gene recombinations. Understanding these recombination events aids in profiling the clonality of the LCLs in HPRC and allows one to distinguish variations driven by V(D)J recombination from germline SVs. We focused on the IGH locus and did not analyze off-target recombinations (recombinations outside of IG loci), although we hypothesize that cryptic RSSs might drive additional rearrangements and thus complicate the inference of germline SVs. Further, we showed specific ways in which somatic recombination events led to assemblies that were either fragmented or failed to represent the germline sequence. Given our understanding of the V(D)J recombination mechanism and the population diversity in the IGH locus, we contend that IGLoo will be an important tool to improve personalized assemblies, especially since it recovers IGH gene segments that would be missed by standard assembly tools.

We found that *de novo* assembly of preprocessed reads improved the representation of the D gene locus while also resulting in some missing J genes. Although the number of D genes gained is greater than the number of J genes lost, there remains a trade-off between the two. Reference-guided assembly appears to recover both D and J genes without this compromise. However, we observed that the reference-guided assembler MaSuRCA occasionally favors the reference genome over *de novo*-assembled contigs, especially when structural differences exist between them. Therefore, it is crucial to construct a personalized reference genome that closely resembles the sample. IGLoo constructs this personalized reference genome based on common SVs reported by Rodriguez et al.^3^ However, we also noted SVs that deviate from these common ones. In such cases, IGLoo cannot generate a reference genome that includes these special SV types, presenting a challenge for MaSuRCA to successfully assemble the region. In the future, as more IG SVs are profiled and cataloged, IGLoo will be able to generate a more accurate personalized reference genome.

In the future, it will be also important to study applicability of the method to IG light-chain loci and examine other somatic recombination events within the IGH locus, e.g., class switch recombination events involving deletions among the constant genes, which we did not address. However, more work and customization will be needed to handle the particulars of these loci and events.

The IGLoo reassembly pipeline serves as a workaround for assemblers that are not designed to handle somatically recombined haplotypes. Hifiasm was designed to assemble phased assemblies based on trio data. However, it does not take into account the fact that parental data can undergo V(D)J recombination, resulting in unbalanced coverage in the IGH locus. The substantial read depletion in the IGH locus also poses a significant challenge to Hifiasm’s *de novo* assembly approach. While MaSuRCA handles the read depletion issue more effectively in the dataset, it lacks the ability to provide a phased assembly. Transferring Hifiasm’s phased results to MaSuRCA partially addresses this issue. Nevertheless, there may still be instances of phasing errors or over-correction by MaSuRCA. A specialized assembler tailored for the IG loci, which considers parental data and addresses the read depletion issue within the locus, could potentially yield more accurate IGH assembly results.

The IGLoo modules are specifically designed for analyzing HiFi datasets and conducting assembly based on HiFi reads. However, the IGLoo --read module can potentially be adapted to handle pair-end short-read data. By examining the aligned positions of both ends of a read pair, a similar analysis to split alignment of HiFi reads can be applied. On the other hand, detecting non-canonical recombination events would not be as straightforward as with HiFi reads. Full non-canonical recombination events likely cannot be captured by a single read pair, requiring some form of inference similar to those used in transcriptome analysis. Another potential challenge for short reads is the closely spaced nature of the J genes in the IGH locus as the distance between two consecutive J genes does not exceed 400 bp. If the non-sequenced insert of the pair-end read falls within the J locus, determining which J gene contributes to the recombination event can be ambiguous and may necessitate inference techniques. The IGLoo --read module can technically be applied to Oxford Nanopore Technologies (ONT) long-read data. Therefore, we provide a --nanopore option to accommodate ONT input data for profiling the recombination events in cell lines. However, we do not recommend use of IGLoo --ReAsm module for ONT data until further validation had been conducted.

Profiling and assembling IG loci based on non-LCL sources (e.g., PBMCs) could be considered a less challenging approach for constructing references for IG loci. However, since LCLs continue to be widely used and abundant in existing studies, IGLoo offers valuable insights for researchers, aiding in the comprehension of IGH locus diversity. IGLoo reconstructs the IGH locus, which is often overlooked in standard assembly pipelines. Although the resulting assembly may not be perfect, it represents a significant step closer to obtaining the best possible results from cell-line data.

### Limitations of the study

IGLoo was designed for HiFi read dataset. While we provide an ONT option for IGLoo --read, we do not recommend using the IGLoo --ReAsm module for ONT data until further validation is conducted. Although IGLoo --ReAsm recovers IGH genes from LCL-based assemblies, the reassembled locus often does not fully revert to its germline form, especially when some regions of the IGH locus are completely lost in the cell line.

## Resource availability

### Lead contact

Requests for further information and resources should be directed to and will be fulfilled by the lead contact, Ben Langmead (langmea@cs.jhu.edu).

### Materials availability

This study analyzes publicly available data and does not generate new reagents.

### Data and code availability

• The personal assemblies, HiFi raw read, and read alignment of the 47 HPRC samples, and the samples’ parental Illumina read and read alignment, are downloaded from the public HPRC S3 bucket https://s3-us-west-2.amazonaws.com/human-pangenomics/index.html?prefix=working/HPRC/ and https://s3-us-west-2.amazonaws.com/human-pangenomics/index.html?prefix=working/HPRC_PLUS/.
• The software of IGLoo is available at https://doi.org/10.5281/zenodo.15048412 and https://github.com/maojanlin/IGLoo under the MIT license.
• Any additional information required to re-analyze the results reported in this study is available from the lead contact upon request.

## Acknowledgments

We thank Justin Wagner and Justin Zook of the Genome In A Bottle project for their advice. We thank Kuan-Hao Chao for advice on genome assembly, and we thank Mohsen Zakeri and Sam Kovaka for their help in running the experiments. We are grateful to Corey T. Watson for useful comments.

M.-J.L. and B.L. were supported by 10.13039/100000057NIGMS grant R35GM139602 and 10.13039/100000051NHGRI grant R01HG011392 to B.L.

This work was carried out at the Advanced Research Computing at Hopkins (ARCH) core facility (rockfish.jhu.edu), which is supported by the 10.13039/100000001National Science Foundation (NSF) grant number OAC 1920103.

## Author contributions

M.-J.L. and Y.S. designed the method. M.-J.L. wrote the software and performed the experiments. M.-J.L. wrote the manuscript. All authors edited and approved the final manuscript.

## Declaration of interests

B.L. is the Principal at InOrder Labs LLC.

## Declaration of generative AI and AI-assisted technologies in the writing process

The authors used ChatGPT to refine the language and clarity of this work. Afterward, the authors reviewed and edited the content as needed and take full responsibility for the content of the final publication.

## STAR★Methods

### Key resources table

**Table:** 

REAGENT or RESOURCE	SOURCE	IDENTIFIER
**Deposited data**		

Human Reference Genome GRCh37	Church et al.^21^	http://www.ncbi.nlm.nih.gov/projects/genome/assembly/grc/human/
Human Reference Genome GRCh38	Church et al.^22^	http://www.ncbi.nlm.nih.gov/projects/genome/assembly/grc/human/
Human Reference Genome T2T-CHM13	Nurk et al.^23^	https://s3-us-west-2.amazonaws.com/human-pangenomics/T2T/CHM13/assemblies/analysis_set/chm13v2.0.fa.gz
Custom IGH reference genome	Rodriguez et al.^3^	http://immunogenomics.louisville.edu/immune_receptor_genomics/current/reference.fasta

**Software and algorithms**		

IGLoo	This paper	https://doi.org/10.5281/zenodo.15048412 https://github.com/maojanlin/IGLoo
Minimap2	Li et al.^20^	https://github.com/lh3/minimap2
yak	Cheng et al.^16^	https://hifiasm.readthedocs.io/en/latest/trio-assembly.html
hifiasm	Cheng et al.^16^	https://hifiasm.readthedocs.io/en/latest/trio-assembly.html
gAIRR-suite	Lin et al.^25^	https://github.com/maojanlin/gAIRRsuite
MaSuRCA	Zimin et al.^17^	https://github.com/alekseyzimin/masurca
BWA MEM	Li et al.^28^	https://github.com/lh3/bwa
IGenotyper	Rodriguez et al.^14^	https://github.com/oscarlr/IGenotyper
BWA MEM	Li et al.^28^	https://github.com/lh3/bwa

**Other**		

Sequence data from HPRC year 1 samples	Wang et al.^11^	https://s3-us-west-2.amazonaws.com/human-pangenomics/index.html?prefix=working/HPRC_PLUS/ https://s3-us-west-2.amazonaws.com/human-pangenomics/index.html?prefix=working/HPRC/

### Method details

#### Profiling V(D)J recombination events using HiFi reads

##### Split alignments and deletions

IGLoo --read accepts a HiFi read file (FASTA/FASTQ) or a read alignment file (BAM/CRAM) as input. When processing alignment file, IGLoo --read extracts the read aligned to IGH locus with samtools view and the BED file specified the IGH locus on the reference genome. When processing a read file, IGLoo --read uses minimap2 to align the reads, then extracts the IGH-locus alignments.

IGLoo --read then analyzes the read alignments, starting from the leftmost J gene to the rightmost D gene. Reads providing evidence of a V(D)J recombination event will typically align in a split fashion across two or more locations on the reference genome. We refer to the distinct portions of the read that align contiguously as “segments.” IGLoo --read identifies split alignments and counts such an alignment as “meaningful” if at least one of the junctions in the split alignment is near the RSS of any V, D, or J gene. Our analysis on HPRC data indicates that the majority of the split sites (93%) fall within 50 bp of an RSS, so we defined the split alignments as sufficiently close if they are within 50 bp apart from RSSs of any IGH genes (Figure S2).

Additionally, IGLoo --read examines the deletions within each “segment”. In cases of non-canonical V(D)J recombination events between D genes, two consecutive D genes may be close enough that the aligner represents the event as a deletion rather than a split alignment. IGLoo --read analyzes each alignment’s CIGAR string, identifying a long deletion between two D genes as indicating a recombination event. Due to the repetitive nature of the D gene locus, the aligner may make different decisions for different reads about where exactly to place the deletion. Hence, the split sites of the deletions may not necessarily be close to an RSS. Therefore, for recombination events between two D genes, we set a loose requirement that the length of deletion be within 50 of the distance between the two relevant D genes.

##### Multiple references

To counter reference bias in the IGH locus, we use three reference genomes: GRCh37, GRCh38, and T2T-CHM13. Each reference has some IGH gene segments not shared by the others. For example, the IGH genes *IGHV1-8* and *IGHV3-9* are common in the population but exist only in GRCh37 and not the others. GRCh38 carries the gene locus from *IGHV4-30-2* to *IGHV4-31*, not present in the other two. Only T2T-CHM13 carries the duplication of gene locus from *IGHV2-70D* to *IGHV1-69D* (Figure S14).

Each extracted read is aligned to all three reference genomes. Of the three choices, IGLoo --read picks the one that best fits the reference genome and its annotation. For instance, an alignment on one reference genome is preferred if all of its split sites are close to RSSs in that reference.

IGLoo --read then classifies each “split” alignment to the kind of event that it supports. An alignment is classified as supporting a “canonical recombination event” if it has a single split with split sites situated near the RSSs of the respective genes. The event is classified as a complete V(D)J recombination if one segment aligned to a J gene and the other segment aligned to a V gene. Otherwise, the event is classified as a D-J recombination if the segments aligned to a J gene and a D gene. Note that for complete V(D)J recombination events, the portion of the D gene present in the read is too short to induce the aligner to make two separate splits on either side of the D gene. As a result, a complete V(D)J event will join one J gene to one V gene, with the D gene sequence appearing on one of the segments, or possibly soft clipped in the alignment. An alignment is classified as supporting a “non-canonical” event in two scenarios. Firstly, if an alignment is split once, with one segment aligned to a D gene and the other aligned to a V gene, it indicates evidence of a V-D recombination utilizing different D gene to the D-J recombination. Hence, it is an evidence of a multiple-D-gene event, or V-D only partial recombination. Alternatively, if the read is split into multiple segments, it suggests a “non-canonical” event involves multiple recombinations or additional inversion.

##### Analyzing the personal assemblies

To analyze the quality of the IGH locus in a personalized assembly, IGLoo --asm first runs gAIRR-annotate^25^ on the assembly. gAIRR-annotate uses BWA MEM^28^ to align documented IGH genes/alleles from the IMGT database to the assembly, reporting the closest genes/alleles and their positions on the assembly. IGLoo --asm then evaluates the assembly based on the distribution of IGH genes on each contig. With the information, IGLoo --asm reports the artifacts of the IGH locus, identifies missing IGH gene segments, and pinpoints the breakpoints and V(D)J junctions in the assembly for assembly quality assessment (Figure 1B).

Outside of the main IGH locus on chromosome 14, there are also many “orphon” genes distributed across the genome. These are possible pseudogenes that are homologous to the main IGH locus. Though they share sequence similarity to IGH gene segments, our focus is solely on the main IGH locus, and we do not aim to improve assembly quality at orphon sites. We therefore filtered out orphons as well as any IGH gene segments with more than 15 mismatches compared to the documented genes, which are likely non-functional genes. IGLoo --asm then filtered out the contigs containing only these genes and retaining the contigs with functional IGH gene segments in the downstream analysis.

IGLoo --asm provides a report on the IGH gene structure, as illustrated in Figure 5E. However, it’s worth noting that the output from gAIRR-annotate, which is solely based on sequence, does not distinguish between duplicated gene pairs such as *IGHV3-23* and *IGHV3-23D*, *IGHV1-69* and *IGHV1-69D*, or *IGHV2-70* and *IGHV2-70D*. To improve interpretability, IGLoo --asm correctly assigns duplicated genes to their respective names. The process involves first tallying the occurrences of identical genes, then assigning duplicated genes with “D” in their name only if multiple instances are detected within the contig. While there are other types of duplicated genes in the IGH locus, they can be easily differentiated by considering their neighboring genes.

After the filtering and gene assignment steps, IGLoo --asm counts the number of contigs and finds the large scale artifacts in the assembly. We classified the artifacts into two main types: “V(D)J junction” and “breakpoint”. The V(D)J junction does not cause the contig to become fragmented but does cause the the loss of IGH gene segments. We detect the V(D)J junction by scanning the IGH gene segments on each contig. If two IGH gene segments from different loci, i.e., V and J loci or D and J loci, are located next to each other within 10 kbp, the connection is considered a V(D)J breakpoint. The only D gene, *IGHD7-27*, which lies within 10 kbp of the J genes, is handled as a special case. The breakpoints correspond to situations where two contigs cannot be connected into one, fragmenting the assembly. The relation between the two contigs can further be categorized into “disjoint”, “overlap”, or “duplication” as depicted in Figure S10. To collect the breakpoints, we first sort the contigs according to their relative position on IGH locus, then check and compare the IGH gene segments on the boundary of the nearby contigs.

##### Reassembly to improve germline assembly of IGH locus

IGLoo --ReAsm performs a series of steps to reassemble the genome to better capture the original germline sequence, rather than the somatically recombined sequence. The workflow is shown in Figure 1C. IGLoo --ReAsm first preprocesses the HiFi reads, splitting the reads with recombination evidence, and duplicating reads in IGHD and IGHJ regions in order to mitigate the evidence of somatic recombination from the original data. Preprocessed reads are then given to hifiasm,^16^ along with k-mers counts from sample’s parents. Hifiasm then uses trio binning^29^ to generate a phased personal assembly. IGLoo --ReAsm then checks the contigs to infer the allele type of some known SVs in the paternal and maternal haplotypes. Based on these, IGLoo --ReAsm generates both paternal and maternal personalized reference haplotypes. The personalized haplotypes, combined with the hifiasm contigs and preprocessed reads, are then given to the reference guided assembler MaSuRCA to generate high quality IGH assembly.^17^ Finally, IGLoo --ReAsm masks out the regions in the high-quality assembly to eliminate any regions whose sequence came only from the reference sequence provided to MaSuRCA, and which were not influenced by the HiFi read sequences.

##### Preprocessing the HiFi reads

This module consists of two steps. The first step filters out read-level evidence originating from recombined portions of the somatic haplotype. Whether a read carries evidence for a somatic event was determined earlier in the IGLoo --read analysis of the split-mapped reads. Consequently, we partitioned the reads into two or more segments based on how many segments their alignment on the reference genome. This effectively eliminates evidence from the somatic haplotype i.e., the V(D)J junction in the read sequence, without discarding the whole read sequence. Note that IGLoo --read does not consider the non-reference connection between two V genes to be somatic V(D)J recombination because this could be evidence of SVs in the sample. Although somatic hypermutations can be present in the reads, IGLoo --ReAsm does not attempt to correct these as they generally will not affect the structure of the assembly. Correction of somatic hypermutations can also falsely remove real polymorphisms in the sample.

The second step performs selective enrichment of reads carrying germline evidence in key loci. Specifically, any read segments mapping to the J gene locus, D gene locus, or the region between J and D genes, are duplicated, yielding a second copy of each. This roughly doubles the depth of the germline sequence representation in the region, which often suffers from read depletion due to the V(D)J recombination events in the cell line. The enrichment step increases the chance that the assembler favors the *de novo* germline sequences in its reconstruction. Note that the enriched reads are used exclusively for *de novo* assembly. In later stages, IGLoo --ReAsm switches back to using the partitioned, but unenriched, read data.

##### Hifiasm draft assembly

The preprocessed and enriched reads are *de novo* assembled with hifiasm (Figures 1C and 2.) In this version of IGLoo --ReAsm, parental short reads are required for the trio-binning mode of hifiasm to improve the contiguity of the assembly. When parental data are not available, users can use hifiasm’s standard mode, treating the primary assembly and secondary assembly as the “paternal” and “maternal” haplotype assemblies for the following stages.

The phased contigs assembled by hifiasm are then used to infer the personal IGH reference (STAR methods: Personalized reference genome) and serve as input scaffolding materials of MaSuRCA (STAR methods: Reference-guided assembly with MaSuRCA)

##### SV allele type and contig reconcile

We genotype the common SVs in the contigs of each parental assembly. We considered both the IMGT database and the findings of Rodriguez et al.^3^ and constructed the following list of common SVs.

(1) deletion from *IGHD2-8* to *IGHD3-3.*
(2) deletion of *IGHV7-4-1.*
(3) complex haplotype of *IGHV3-64D* and *IGHV5-10-1*, or *IGHV1-8* and *IGHV3-9.*
(4) deletion of *IGHV3-23D.*
(5) deletion from *IGHV4-30-2* to *IGHV3-33.*
(6) deletion from *IGHV4-38-2* to *IGHV1-38-4.*
(7) deletion from *IGHV2-70D* to *IGHV1-69D*

We type the SVs by first calling gAIRR-annotate to collect the IGH gene segments on the *de novo assembled* contigs. If one contig carries the genes neighboring the two ends of a deletion, but not the genes in the deletion, the contig is considered to support that deletion. On the other hand, if the contig carries the genes inside the deletion region, the contig does not support the deletion. If a contig does not cover the region, the allele type is “unknown” for that SV in the contig. Previous work used the term “complex” to describe an SV involving two haplotypes, one carrying *IGHV3-64D* and *IGHV5-10-1*, and the other carrying *IGHV1-8* and *IGHV3-9*. We genotype this SV by searching for genes that are unique to one of the two haplotypes. If the contig does not carry any of the four unique genes, or carries unique genes from both haplotypes, then the allele type is “unknown”.

The IGLoo --ReAsm checks if there are SV allele type conflicts within paternal contigs or maternal contigs. If there are conflicted contigs, IGLoo --ReAsm tries to move the conflicted contig to another parental group. If the conflict can be simply reconciled by moving the conflicted contigs around, IGLoo --ReAsm report reconciliation successful and move to the next stage. Otherwise IGLoo --ReAsm report reconciliation fail and request manual reconciliation.

##### Personalized reference genome

To enable more accurate reference-guided assembly, we generate a personalized reference genome tailored to each individual’s SV haplotypes. The personalized genome is constructed by stitching together sequences from five existing IGH reference representations: T2T-CHM13, GRCh37, GRCh38, a “Custom” IGH reference genome described in,^3^ and the *de novo* assembled sequence of HG02148’s paternal haplotype. Table 1 lists the selected references for the 7 common SVs described in STAR methods: SV allele type and contig reconcile. For deletions, i.e., all except complex SV number 3, the default haplotype is set to the one without deletion, using sequences from T2T-CHM13 and the “Custom” reference. For references representing deletion haplotypes, we use sequences from HG02148, GRCh37, GRCh38, and T2T-CHM13. In the case of the complex SV, the haplotype carrying *IGHV1-8* and *IGHV3-9* is from T2T-CHM13, while the haplotype carrying *IGHV3-64D* and *IGHV5-10-1* is from GRCh38.

**Table 1:** The sequence of choices made when building a personalized reference genome according to SV allele type

SV ID	Default hap	Alternative hap
	Without deletion	With deletion
1	CHM13	HG02148
2	CHM13	GRCh37
4	Custom	CHM13
5	CHM13	GRCh38
6	Custom	CHM13
7	CHM13	GRCh38
3	CHM13 (*IGHV1-8*)	GRCh38 (*IGHV3-64D*)

“Custom” is the custom IGH reference genome from Rodriguez et al.^3^ HG02148 is the *de novo*-assembled paternal haplotype of HG02148. SVs 1, 2, and 4–7 represent simple deletions. SV3 is a complex structural variant involving two distinct haplotypes, as indicated by the IGHV gene names in parentheses.

When stitching the sequences together, we used T2T-CHM13 as the backbone for non-SV regions. Customized sequences are extended to include the closest nearby IGH gene segments. Using the SV allele types identified from the previous stage, a personalized reference genome for each parental haplotype is created.

##### Reference-guided assembly with MaSuRCA

We use the personalized reference as the guided reference of MaSuRCA, the *de novo* assembled contigs as the scaffolding materials. MaSuRCA first patches the contigs with the reference genome as the draft assembly. This causes gaps between contigs to be filled with sequence from the reference. We then use yak triobin to separate the HiFi reads into paternal and maternal groups. Reads that could not be confidently assigned to one haplotype were included in both groups to help polish their assemblies.

##### Masking false-positive regions

Since the output contains some “patched” sequences taken from the reference, we filtered out regions without support from either contigs or reads. MaSuRCA uses the “capitalized nucleotide” to represent region in the assembly support by contigs or polished by the reads. We first discard MaSuRCA contigs not supported by any *de novo* assembled contigs. Then for the remaining MaSuRCA contigs, we align the processed reads to them. We use samtools depth -a -g 0x100 -J to check which regions are covered by read alignments. For regions without any coverage, we mask the region with “N”s.

##### Experiments perform on HPRC samples

We applied the IGLoo analysis pipeline to the 47 individuals of the HPRC year 1 release. Though not in our released software, we used GNU parallel^30^ in our experiments.

In the Hifiasm trio-binning stage of IGLoo --ReAsm (STAR methods: Hifiasm draft assembly), most of the parental data of the 47 samples are already aligned to GRCh38. Only the parental data of HG002, HG005, and NA21309 were not already aligned, and instead were provided as raw reads. For these, we used BWA MEM to align the reads to GRCh38. Reads aligning to the IGH locus were then extracted using samtools view. Only these reads were provided to the hifiasm trio-binning method, saving time and space compared to if all reads had been provided to hifiasm.

When building the personal reference of the HPRC samples (STAR methods: SV allele type and contig reconcile), three samples HG01891, HG03453, and NA18906 could not be automatically reconciled, as discussed in Results. We manually reconciled these haplotypes.

In the polishing stage (STAR Methods: Reference-guided assembly with MaSuRCA), 10 individuals had coverage histograms of at least one haplotype that were shifted lower, and so required that we set this parameter to a lower value. In these cases, we set this parameter to 2.

##### Evaluation on the IGLoo assemblies

To evaluate the quality of a IGLoo reassembled IGH assembly, we first cropped the contigs containing IGH gene segments from the HPRC assembly to serve as the baseline for comparison. During cropping, we extended the region by 100 kbp on the telomere end beyond the last IGHV gene and by 376 kbp on the centromere end beyond the first IGHJ gene to include the constant genes in the IGH locus. Using the yak trioeval tool, we assessed phasing quality by calculating switching error rates. yak trioeval assigns paternal or maternal sites on the assembly based on k-mers collected from parental Illumina short-read data. The switching error rate is defined as the ratio of paternal-to-maternal or maternal-to-paternal switches among all neighboring site pairs in the assembly.

In addition, HPRC provides WGS Illumina short-read alignments for 43 out of 47 individuals. For these samples, we collected reads mapped to the IGH region in GRCh38, along with unmapped reads, and realigned them to the IGLoo assemblies. Alignments with mapping quality below 10 were filtered out. We then analyzed whether these reads with decent mapping quality supported the newly assembled IGH gene segments. First, we determined how many newly assembled IGH gene segments were fully covered. An IGH gene segment is considered fully covered if all of its bases were covered by at least one read. For fully covered IGH gene segments, we further evaluated the edit distance between aligned reads and the assembly. Each base of the IGH gene segment was examined to determine if it had at least one read support. Bases that were covered but not supported, due to mismatches, insertions, or deletions, were summed into the edit distance. Since Illumina short reads were not used during the assembly process, this comparison provides a robust and independent evaluation of the assembly quality.

### Quantification and statistical analysis

The correlation between Simpson’s index and the number of distinct events is assessed with Spearman’s rank correlation coefficient. The Spearman coefficient is computed with the function scipy.stats.spearmanr from SciPy (version 1.10.1). Boxplots and swarm plots in this manuscript are generated using the boxplot and swarmplot functions from the seaborn package (version 0.12.2).

## Supplemental information

Document S1. Figures S1–S14

Document S2. Article plus supplemental information
