# Supplementary material for: IGLoo enables comprehensive analysis and assembly of immunoglobulin heavy-chain loci in lymphoblastoid cell lines using PacBio high-fidelity reads
Source: Cell Rep Methods. 2025 May 1;5(5):101033. doi: 10.1016/j.crmeth.2025.101033 (PMC12146632; doi:10.1016/j.crmeth.2025.101033)
Supplement: Document S1. Figures S1–S14 [file mmc1.pdf]

Cell Reports Methods, Volume *5*

Supplemental information

IGLoo enables comprehensive analysis and assembly of immunoglobulin heavy-chain loci in lymphoblastoid cell lines using PacBio high-fidelity reads

Mao-Jan Lin, Ben Langmead, and Yana Safonova

Supplementary Material

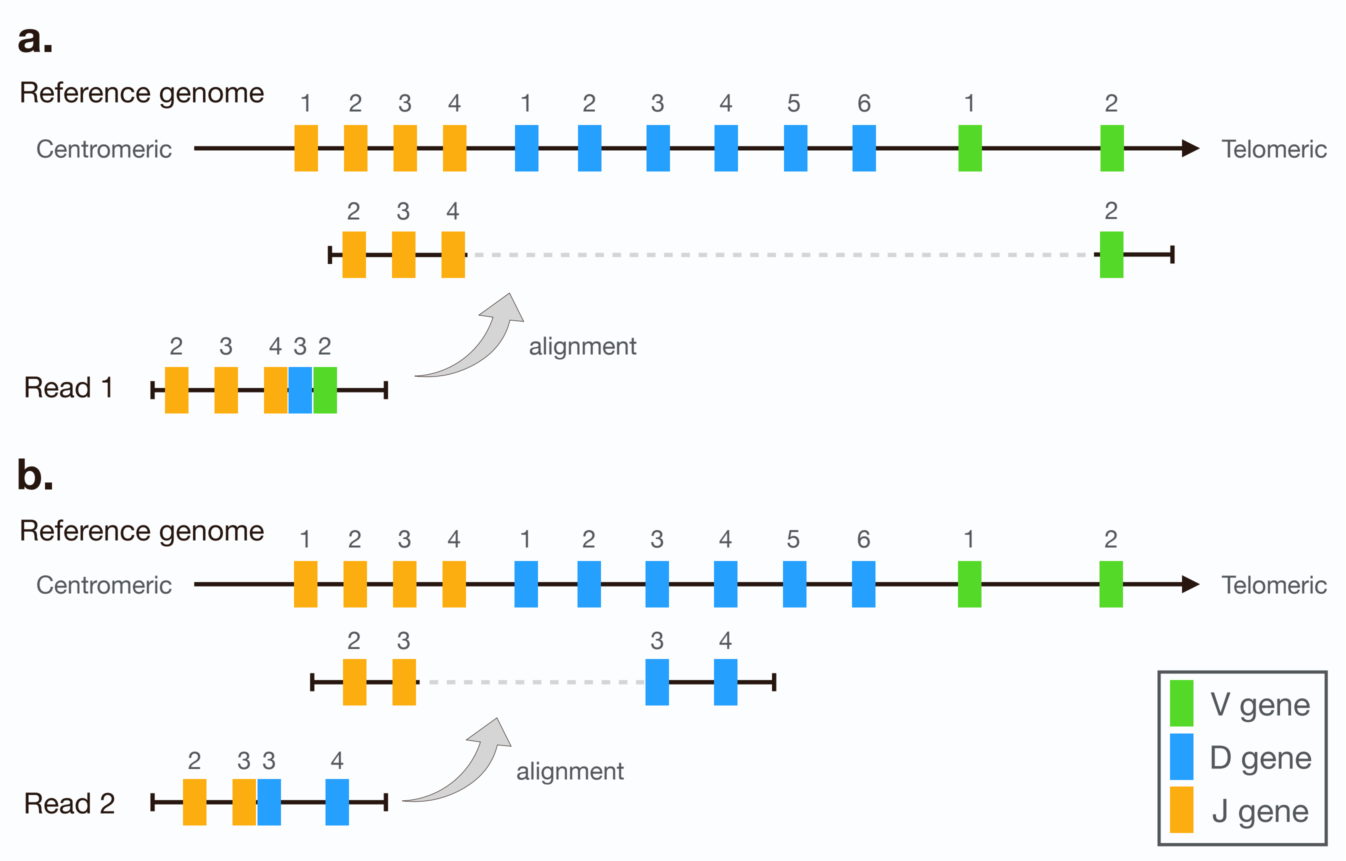

Figure S1:

Figure S1. Schematic examples of HiFi read alignments carrying recombination events mapped to a reference genome

a. Read 1 carries a complete V(D)J recombination. The alignment maps the first half of the read to the J gene locus and the second half to the corresponding V region. The D gene segment within the read is too short for most long-read aligners to map as a distinct segment. **b**. Read 2 carries a D-J recombination event. The alignment maps the first half of the read to the J gene locus and the second half to the corresponding D region. Note that the numbers on the reference genome are illustrative and do not correspond to actual IGH gene identities.

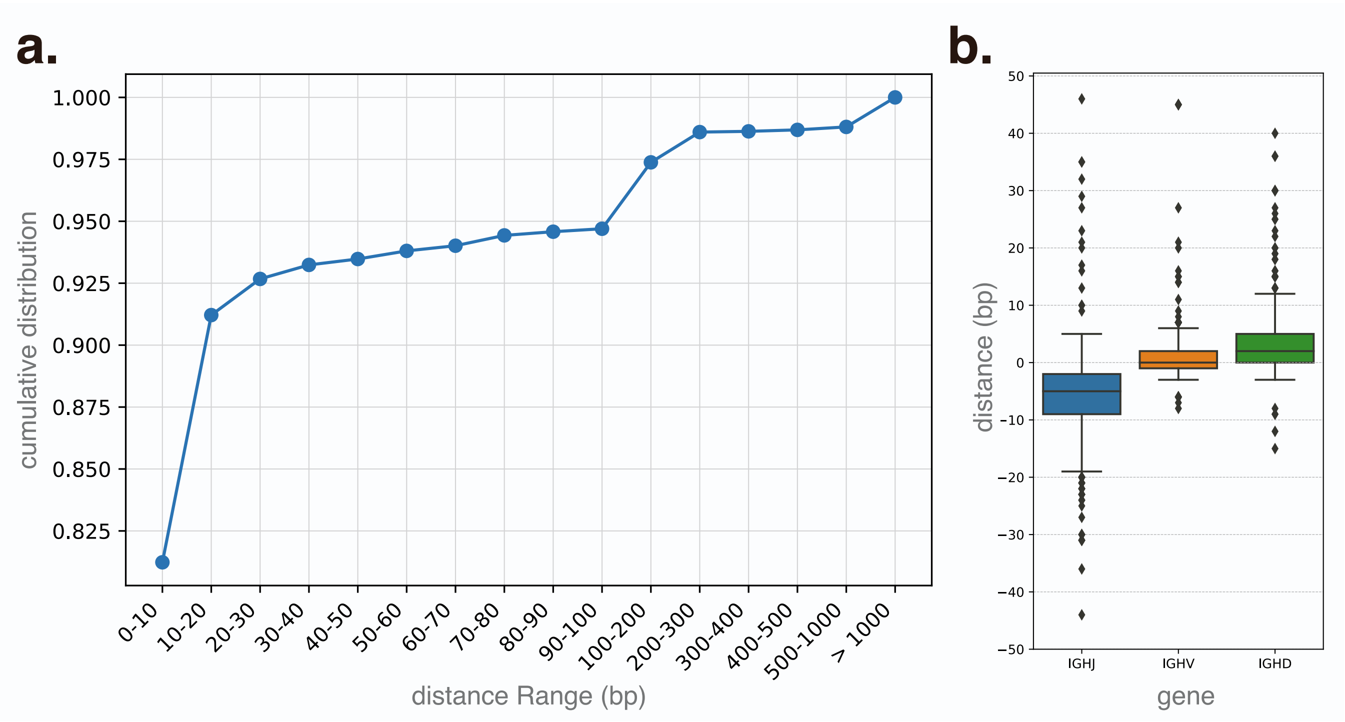

Figure S2. The distribution of the distance of the $3,357$ split site to RSSs

a. the cumulative plot of the distribution of the absolute distance (bp) to the closest RSS. **b.** the distribution of the distance (bp) to RSS stratified by IGHJ, IGHV, and IGHD genes. We only show the cases with distance < 50 bp. The distance of J genes are counted on its $5'$ end, while the distance of V and D genes are counted from their $3'$ end.

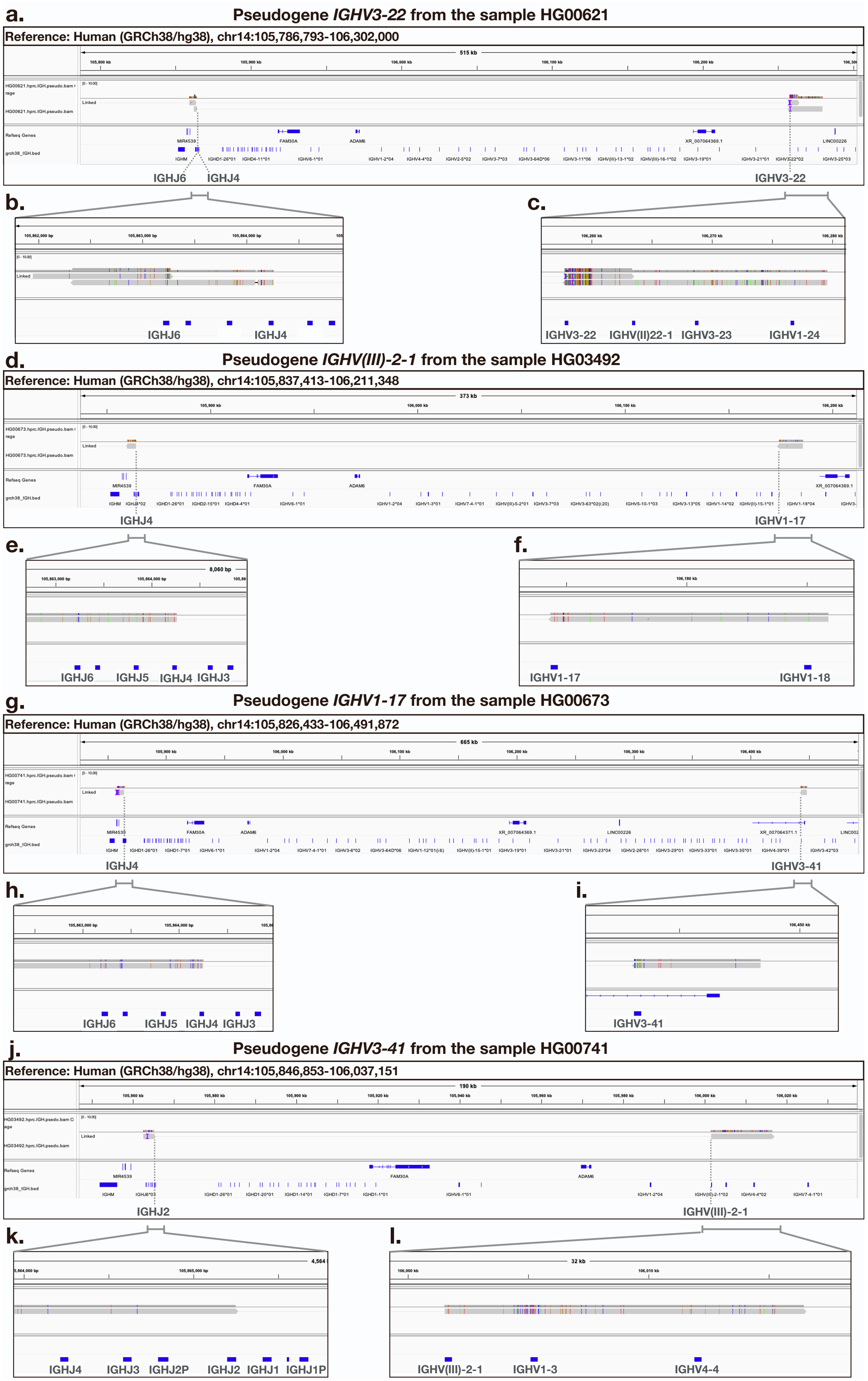
a.
Pseudogene IGHV3-22 from the sample HG00621
Reference: Human (GRCh38/hg38), chr14:105,786,793-106,302,000
IGHJ6
IGHJ4
IGHV3-22
b.
IGHJ6
IGHJ4
c.
IGHV3-22
IGHV(II)22-1
IGHV3-23
IGHV1-24
d.
Pseudogene IGHV(III)-2-1 from the sample HG03492
Reference: Human (GRCh38/hg38), chr14:105,837,413-106,211,348
IGHJ4
IGHV1-17
e.
IGHJ6
IGHJ5
IGHJ4
IGHJ3
f.
IGHV1-17
IGHV1-18
g.
Pseudogene IGHV1-17 from the sample HG00673
Reference: Human (GRCh38/hg38), chr14:105,826,433-106,491,872
IGHJ4
IGHV3-41
h.
IGHJ6
IGHJ5
IGHJ4
IGHJ3
i.
IGHV3-41
j.
Pseudogene IGHV3-41 from the sample HG00741
Reference: Human (GRCh38/hg38), chr14:105,846,853-106,037,151
IGHJ2
IGHV(III)-2-1
k.
IGHJ4
IGHJ3
IGHJ2P
IGHJ2
IGHJ1
IGHJ1P
l.
IGHV(III)-2-1
IGHV1-3
IGHV4-4

Figure S3. The complete V(D)J recombination event using the pseudo-genes *IGHV3-22*, *IGHV(III)-2-1*, *IGHV1-17*, and *IGHV3-41*

a. The read alignment showing complete V(D)J recombination using *IGHV3-22* from the sample HG00621 on the reference genome. **b**. zoom in view of the split-sites around the J genes. **c**. zoom in view of the split-sites around the *IGHV3-22*. **d**. The read alignment showing complete V(D)J recombination using *IGHV(III)-2-1* from the sample HG03492 on the reference genome. **e**. zoom in view of the split-sites around the J genes. **f**. zoom in view of the split-sites around the *IGHV(III)-2-1*. **g**. The read alignment showing complete V(D)J recombination from the sample HG00673 on the reference genome. **h**. zoom in view of the split-sites around the J genes. **i**. zoom in view of the split-sites around the *IGHV1-17*. **j**. The read alignment showing complete V(D)J recombination from the sample HG00741 on the reference genome. **k**. zoom in view of the split-sites around the J genes. **l**. zoom in view of the split-sites around the *IGHV3-41*.

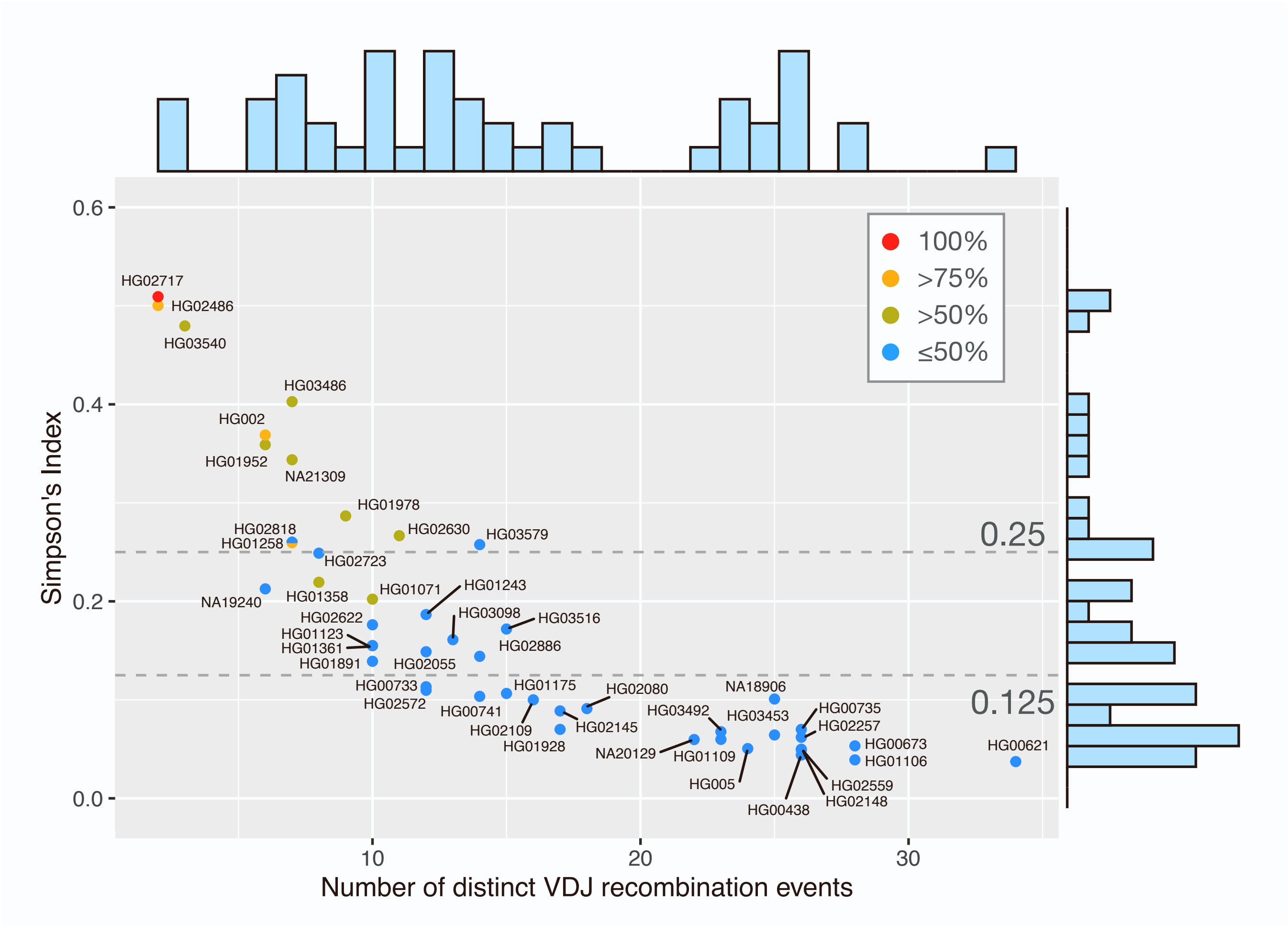

Figure S2:

Figure S4. Comparison of the clonality classification using Simpson's index and using the method from[13]

The position of each sample are calculated as in Result section: Clonality of the cell lines, which considering all the recombination events. The color of each sample represents its clonality evaluation from[13], which considers only complete V(D)J recombination events. The samples are classified into four categories according to the fraction of reads supporting the dominant complete V(D)J recombination event. Since the position of HG02818 and HG01258 are so close, we divided their dots into half to show the two colors.

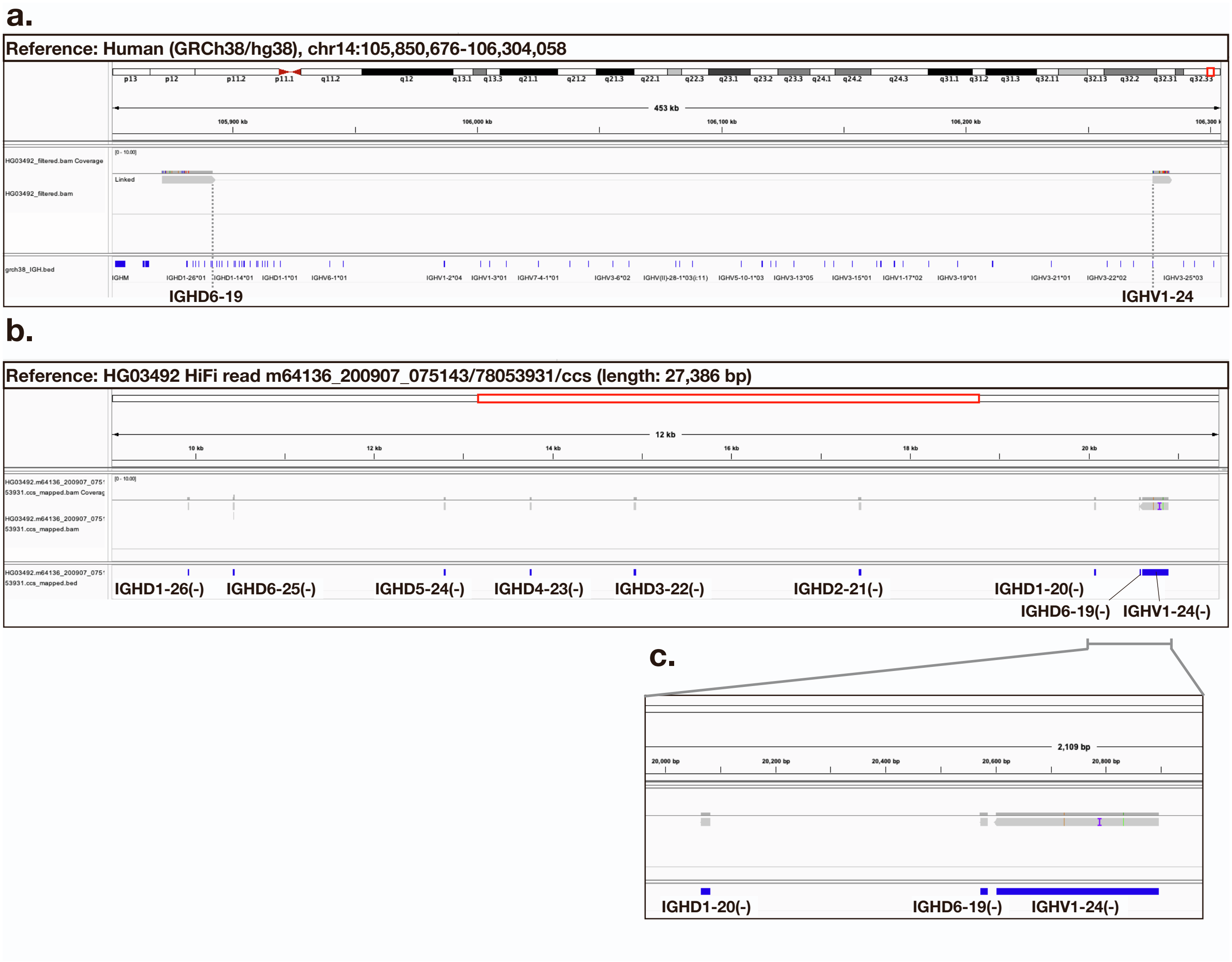

Figure S5. The V-D only partial recombination event we observed in HPRC from individual HG03492

a. The read alignment showing V-D only recombination on the reference genome. The read is split into two, with the break points near the boundary of *IGHD6-19* and *IGHV1-24*. Note that the $3'$ end segments of the read stretch to the position between IGHD and IGHJ loci. **b**, the annotation of one read carried the event. In this IGV screenshot, the HiFi read sequence is served as the reference, and we aligned the IGH gene segments to the read sequence for annotation. The – (reverse) and + (forward) in the parentheses after the gene name indicates the orientation of the alignment. **c**. zoom in view of the recombination site in the read.

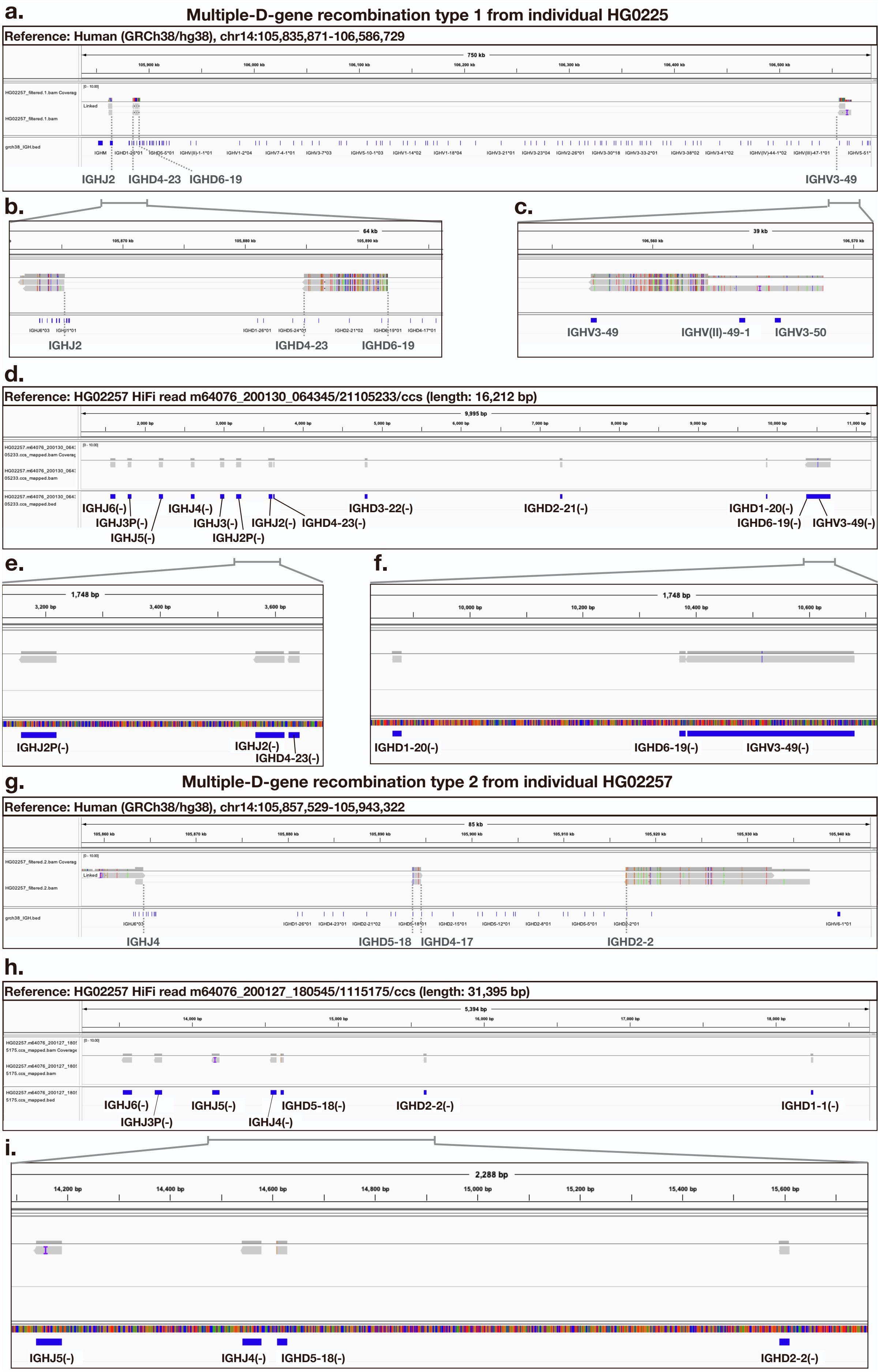

a.
Multiple-D-gene recombination type 1 from individual HG0225
Reference: Human (GRCh38/hg38), chr14:105,835,871-106,586,729
IGHJ2
IGHD4-23
IGHD6-19
IGHV3-49
b.
IGHJ2
IGHD4-23
IGHD6-19
c.
IGHV3-49
IGHV(II)-49-1
IGHV3-50
d.
Reference: HG02257 HiFi read m64076_200130_064345/21105233/ccs (length: 16,212 bp)
IGHJ6(-)
IGHJ3P(-)
IGHJ5(-)
IGHJ4(-)
IGHJ3(-)
IGHJ2P(-)
IGHJ2(-)
IGHD4-23(-)
IGHD3-22(-)
IGHD2-21(-)
IGHD1-20(-)
IGHD6-19(-)
IGHV3-49(-)
e.
1,748 bp
IGHJ2P(-)
IGHJ2(-)
IGHD4-23(-)
f.
1,748 bp
IGHD1-20(-)
IGHD6-19(-)
IGHV3-49(-)
g.
Multiple-D-gene recombination type 2 from individual HG02257
Reference: Human (GRCh38/hg38), chr14:105,857,529-105,943,322
IGHJ4
IGHD5-18
IGHD4-17
IGHD2-2
h.
Reference: HG02257 HiFi read m64076_200127_180545/1115175/ccs (length: 31,395 bp)
IGHJ6(-)
IGHJ3P(-)
IGHJ5(-)
IGHJ4(-)
IGHD5-18(-)
IGHD2-2(-)
IGHD1-1(-)
i.
2,288 bp
IGHJ5(-)
IGHJ4(-)
IGHD5-18(-)
IGHD2-2(-)

Figure S6. The non-canonical multiple-D-gene recombination event from the individual HG02257

a-f: First type of non-canonical multiple-D-gene recombination event from the individual HG02257. **a**. The read alignment shows that the full recombination event has two breakpoints utilizing *IGHD4-23* and *IGHD6-19*. The two D genes paired with *IGHJ2* and *IGHV3-49* respectively. **b**. zoom in view of the breakpoints around J and D genes. **c**. zoom in view of the breakpoint of the V gene. **d**. the annotation of one read carried the event. In this IGV screenshot, the HiFi read sequence is served as the reference. **e.** zoom in view of the D-J junction. **f**. zoom in view of the V-D junction of the read. **g-i**: Second type of non-canonical multiple-D-gene recombination event from the individual HG02257. **g**. The read alignment shows that there is one D-J recombination utilizing *IGHJ4* and *IGHD5-18*. And there is another D-D recombination utilizing *IGHD4-17* and *IGHD2-2*. **h**. the annotation of one read carried the event. In this IGV screenshot, the HiFi read sequence is served as the reference. **i**. zoom in view of the D-J junction and the D-D junction. Notice that the sequence of *IGHD4-17* were deleted in the D-D recombination, so the annotation cannot show its position. However, in the germline IGH, *IGHD5-18* and *IGHD2-2* are far from each other. As a result, there must be a large scale deletion in this read.

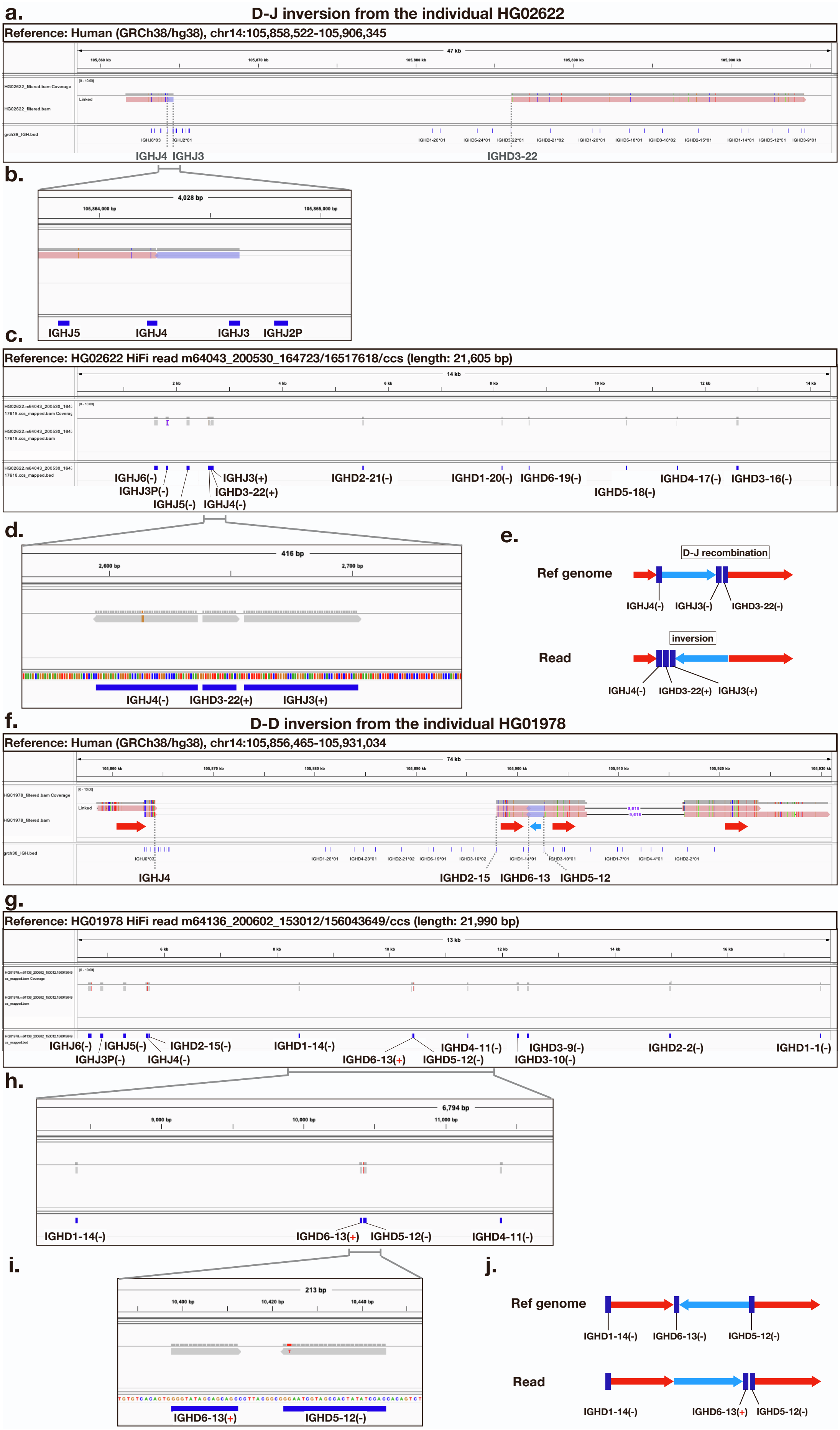
a.
D-J inversion from the individual HG02622
Reference: Human (GRCh38/hg38), chr14:105,858,522-105,906,345
IGHJ4 IGHJ3
IGHD3-22
b.
c.
IGHJ5
IGHJ4
IGHJ3
IGHJ2P
Reference: HG02622 HiFi read m64043_200530_164723/16517618/ccs (length: 21,605 bp)
IGHJ6(-)
IGHJ3P(-)
IGHJ5(-)
IGHJ4(-)
IGHJ3(+)
IGHD3-22(+)
IGHD2-21(-)
IGHD1-20(-)
IGHD6-19(-)
IGHD5-18(-)
IGHD4-17(-)
IGHD3-16(-)
d.
IGHJ4(-)
IGHD3-22(+)
IGHJ3(+)
e.
Ref genome
D-J recombination
IGHJ4(-)
IGHJ3(-)
IGHD3-22(-)
Read
inversion
IGHJ4(-)
IGHD3-22(+)
IGHJ3(+)
f.
D-D inversion from the individual HG01978
Reference: Human (GRCh38/hg38), chr14:105,856,465-105,931,034
IGHJ4
IGHD2-15
IGHD6-13
IGHD5-12
g.
Reference: HG01978 HiFi read m64136_200602_153012/156043649/ccs (length: 21,990 bp)
IGHJ6(-)
IGHJ3P(-)
IGHJ5(-)
IGHJ4(-)
IGHD2-15(-)
IGHD1-14(-)
IGHD6-13(+)
IGHD5-12(-)
IGHD4-11(-)
IGHD3-9(-)
IGHD3-10(-)
IGHD2-2(-)
IGHD1-1(-)
h.
IGHD1-14(-)
IGHD6-13(+)
IGHD5-12(-)
IGHD4-11(-)
i.
IGHD6-13(+)
IGHD5-12(-)
j.
Ref genome
IGHD1-14(-)
IGHD6-13(-)
IGHD5-12(-)
Read
IGHD1-14(-)
IGHD6-13(+)
IGHD5-12(-)

Figure S7. The non-canonical recombination events with D-J inversion and D-D inversion.

a-e: The non-canonical recombination event with an inversion involving J genes from the sample HG02622. **a**. the alignment of the recombination event. Three genes *IGHJ4*, *IGHJ3* and *IGHD3-22* are involved in the full event. **b**. the zoom in view of the inverted sequence between *IGHJ4* and *IGHJ3*. **c**. The annotation of one read carried the event. In this IGV screenshot, the HiFi read sequence is served as the reference, and we aligned the IGH gene segments to the read sequence for annotation. The - (reverse) and + (forward) indicates the orientation of the alignment. **d**. The zoom in view of the point of inversion. Note that the *IGHJ4* is reversed while both *IGHD3-22* and *IGHJ3* are forward. **e**. The whole recombination event can be explained by a D-J recombination between *IGHJ3* and *IGHD3-22*, which generate the sequence in reference genome orientation, and one inversion utilizing the *IGHJ4* and the remaining end of *IGHD3-22*. These two events result in the final read sequence with all the three genes involved situated side by side. **f-j**: The non-canonical recombination with inversion on D genes from the individual HG01978. **f**. the non-canonical recombination event that involved an inversion between *IGHD6-13* and *IGHD5-12*. The orientation of the read alignment is marked with red and blue arrows. The sequence in between the two D genes is inverted. **g**. The annotation of one read carried the event. In this IGV screenshot, the HiFi read sequence is served as the reference, and we aligned the IGH gene segments to the read sequence for annotation. The - (reverse) and + (forward) in the parentheses after the gene name indicates the orientation of the alignment. **h**. the zoom in view of the inverted region on the read sequence. Notice that in germline sequence, *IGHD6-13* is positioned near the middle between *IGHD1-14* and *IGHD5-12*. The inversion flip the *IGHD6-13* next to *IGHD5-12*. **i**. The zoom in view of the junction of the inversion. Notice that the orientation of the two D genes are different. **j**. schematic plot of the inversion event.

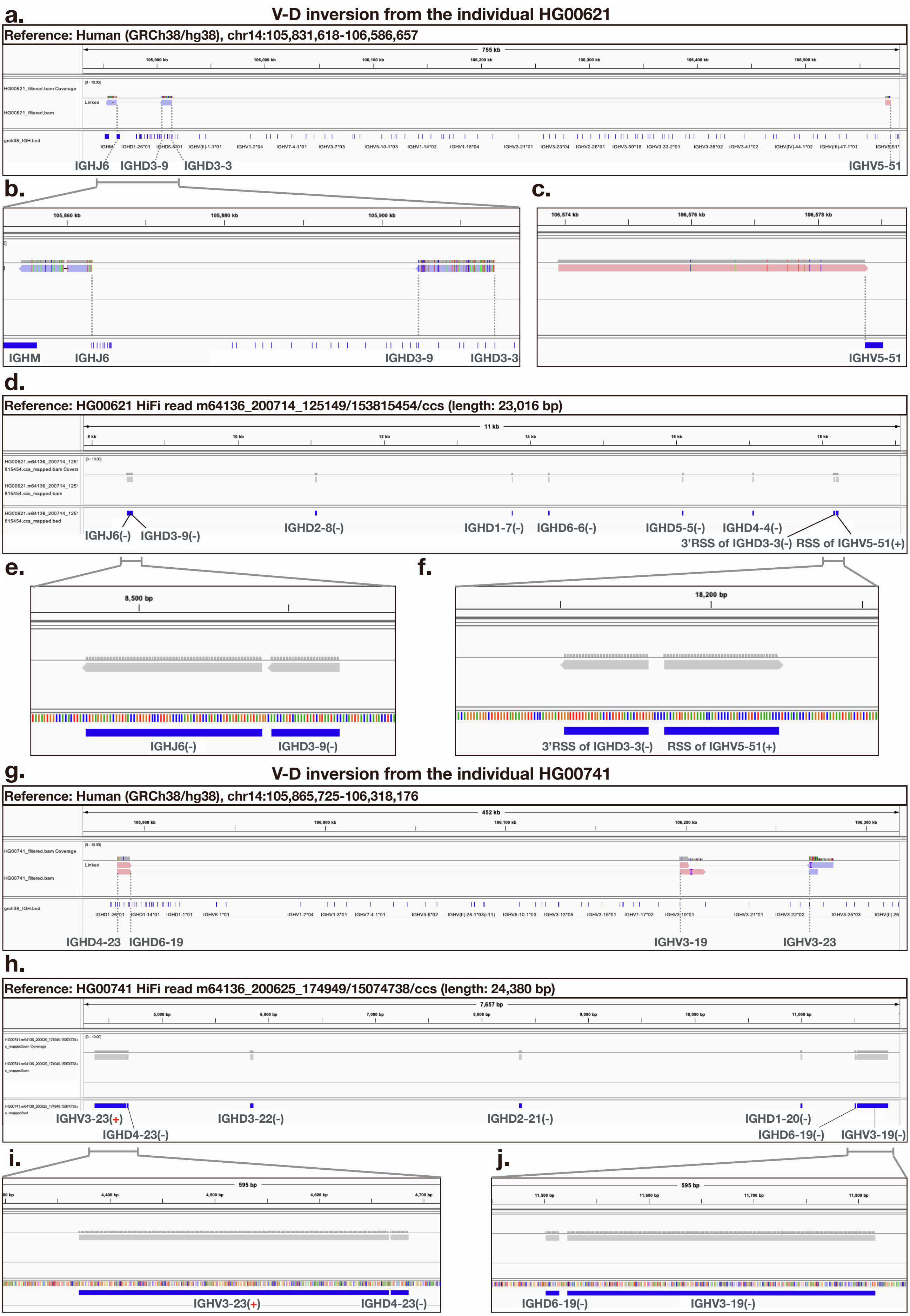
a.
V-D inversion from the individual HG00621
Reference: Human (GRCh38/hg38), chr14:105,831,618-106,586,657
IGHJ6
IGHD3-9
IGHD3-3
IGHV5-51
b.
IGHM
IGHJ6
IGHD3-9
IGHD3-3
c.
IGHV5-51
d.
Reference: HG00621 HiFi read m64136_200714_125149/153815454/ccs (length: 23,016 bp)
IGHJ6(-)
IGHD3-9(-)
IGHD2-8(-)
IGHD1-7(-)
IGHD6-6(-)
IGHD5-5(-)
IGHD4-4(-)
3'RSS of IGHD3-3(-)
RSS of IGHV5-51(+)
e.
IGHJ6(-)
IGHD3-9(-)
f.
3'RSS of IGHD3-3(-)
RSS of IGHV5-51(+)
g.
V-D inversion from the individual HG00741
Reference: Human (GRCh38/hg38), chr14:105,865,725-106,318,176
IGHD4-23
IGHD6-19
IGHV3-19
IGHV3-23
h.
Reference: HG00741 HiFi read m64136_200625_174949/15074738/ccs (length: 24,380 bp)
IGHV3-23(+)
IGHD4-23(-)
IGHD3-22(-)
IGHD2-21(-)
IGHD1-20(-)
IGHD6-19(-)
IGHV3-19(-)
i.
IGHV3-23(+)
IGHD4-23(-)
j.
IGHD6-19(-)
IGHV3-19(-)

Figure S8. The non-canonical recombination events with V-D inversion

a-f: The IGV screenshot of the individual HG00621 with a V-D inversion event. **a**. The split-alignments of the read carrying the inversion event on the reference GRCh38. The event involved a D-J recombination between *IGHJ6* and *IGHD3-9*, and an inversion between *IGHD3-3* and *IGHV5-51*. **b**. The D-J recombination. **c**. The breakend of *IGHV5-51*. **d**. The annotation of the read carried this event. In this IGV screenshot, the HiFi read sequence is served as the reference, and we aligned the IGH gene segments to the read sequence for annotation. The - (reverse) and + (forward) in the parentheses after the gene name indicates the orientation of the alignment. **e**. The D-J junction. Note that the orientation of the two genes are the same. **f**. The inversion breakend between the $3'$ RSS of *IGHD3-3* and the RSS of *IGHV5-51*, where the orientations of the two sequences are reversed relative to each other. **g-j**: The IGV screenshot of the individual HG00741 with the double V-D recombination event. **g**. The split-alignments of two reads carrying this event on the reference GRCh38. The event involved a V-D recombination between *IGHD6-19* and *IGHV3-19*, and another V-D recombination event between *IGHD4-23* and *IGHV3-23*. **h**. The annotation of one read carried the event. In this IGV screenshot, the HiFi read sequence is served as the reference, and we aligned the IGH gene segments to the read sequence for annotation. The - (reverse) and + (forward) in the parentheses after the gene name indicates the orientation of the alignment. **i**. the zoom in view of the 3' end V-D recombination, notice that the *IGHV3-23* is forward but the *IGHD4-23* is reversed. **j**. the zoom in view of the 5' end V-D recombination. Both of the genes are reversed with respect to the read sequence.

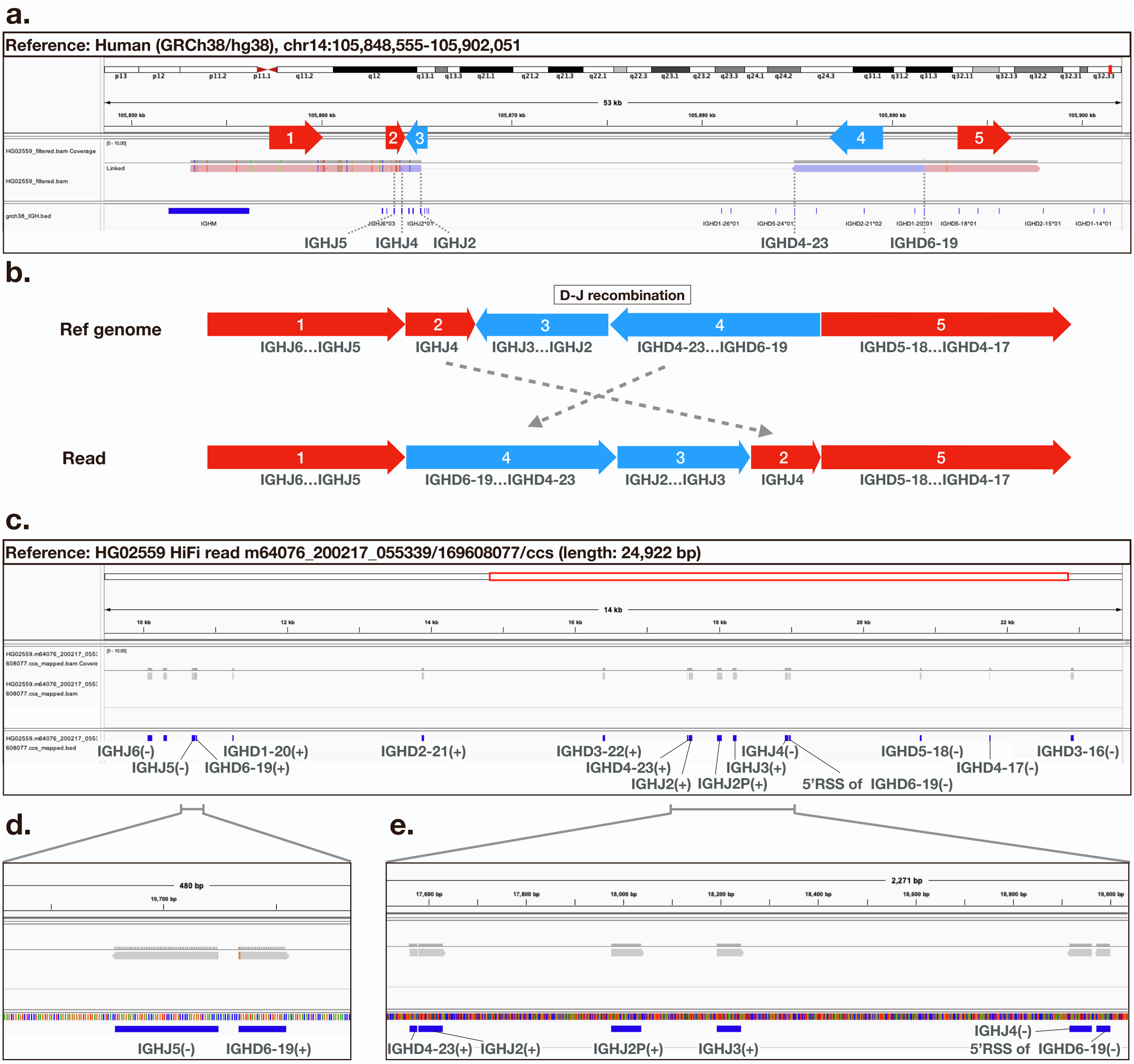

Figure S9. A complex non-canonical recombination event from individual HG02559

a. The read is split into five segments when aligning to reference genome. The orientation of each segment is marked with red and blue arrows. **b**. The comparison of the read sequence in reference genome orientation and in the raw read form. The non-canonical recombination event can be explained by a D-J recombination between *IGHJ2* and *IGHD4-23*, and an additional complex SV. The complex SV is one translocation of the segment marked with 2 and the inversion of the segment marked with 3 and 4. **c**. the annotation of one read carried the event. In this IGV screenshot, the HiFi read sequence is served as the reference, and we aligned the IGH gene segments to sequence for annotation. The − (reverse) and + (forward) indicates the orientation of

the alignment. **d**. The zoom in view of one inversion breakpoint between *IGHJ5* and *IGHD6-19*, note that the orientation of the two genes are different. **e**. zoom in view of another breakpoint of the inversion between *IGHJ3* and *IGHJ4*. Note that the D-J recombination and the translocation boundary between *IGHJ4* and *IGHD6-19*'s flanking sequence are also in the screenshot.

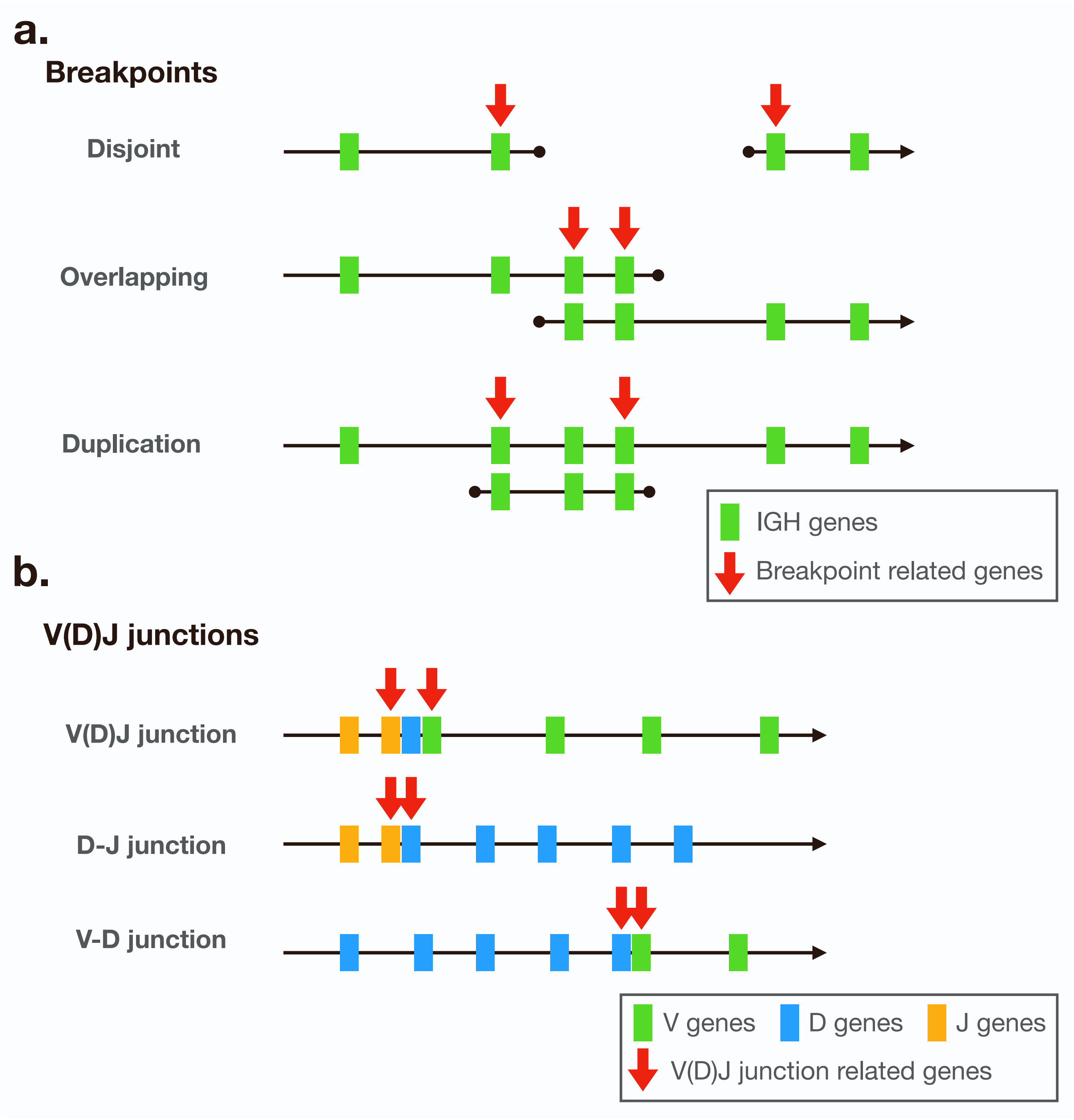

Figure S10. Categories of the breakpoints and V(D)J junctions in IGH locus

a. There are three subtypes of breakpoints: **Disjoint**, the contigs end without overlapping each other. **Overlapping**, the assembler reports two separate contigs but with part of the contig overlapping each other. **Duplication**, one contig is contained in the other contig. **b.** V(D)J junctions are where two IGH gene segments were connected due to recombination events. The non-germline connection can be V(D)J, D-J or V-D depending on the recombination event.

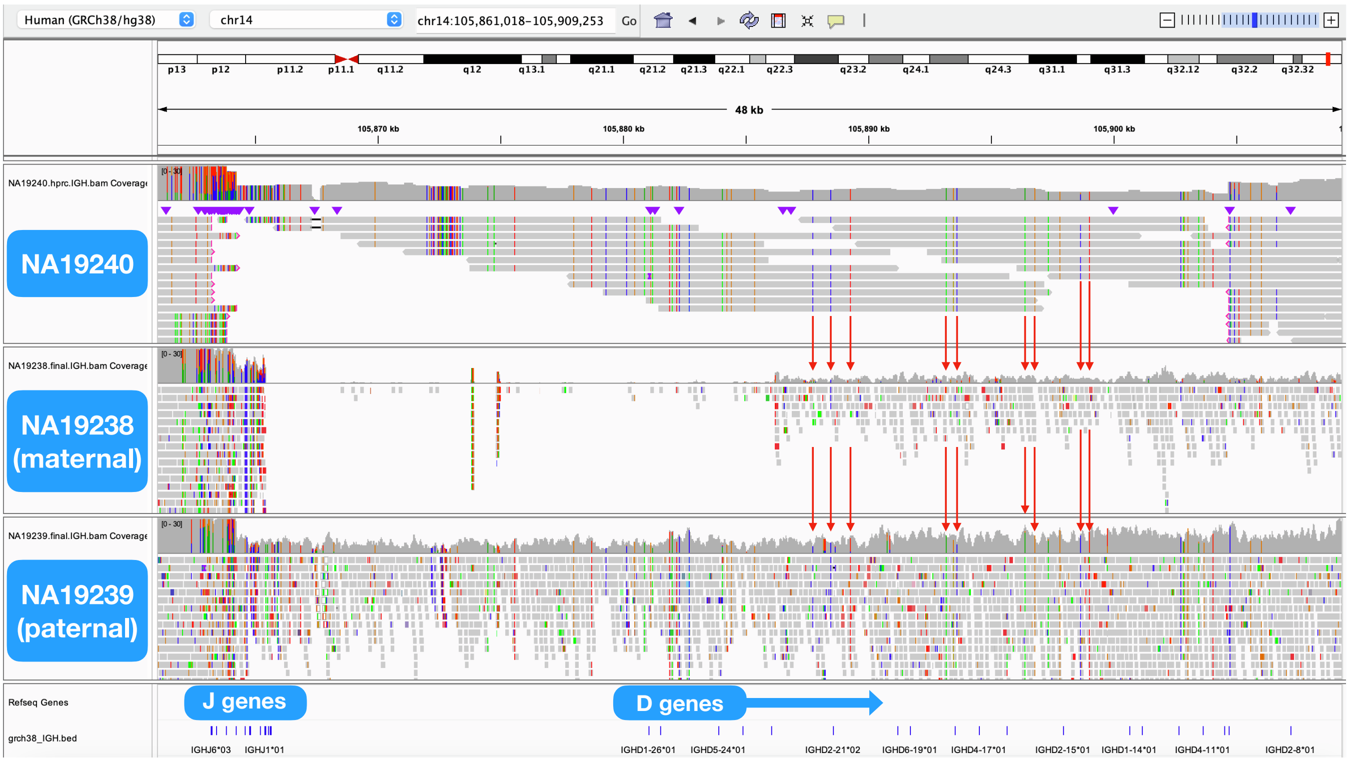

Figure S11. Read alignment of the NA19240 trio family at the IGHJ and IGHD loci

The IGV screenshot of the read mapping on the IGHD/IGHJ loci of sample NA19240 and its maternal (NA19238) and paternal (NA19239) data. Notably, the D gene locus in NA19240 exhibits haploid-like evidence. The red arrows highlight the SNP evidence in the NA19240 haplotype that closely matches the paternal (NA19239) data.

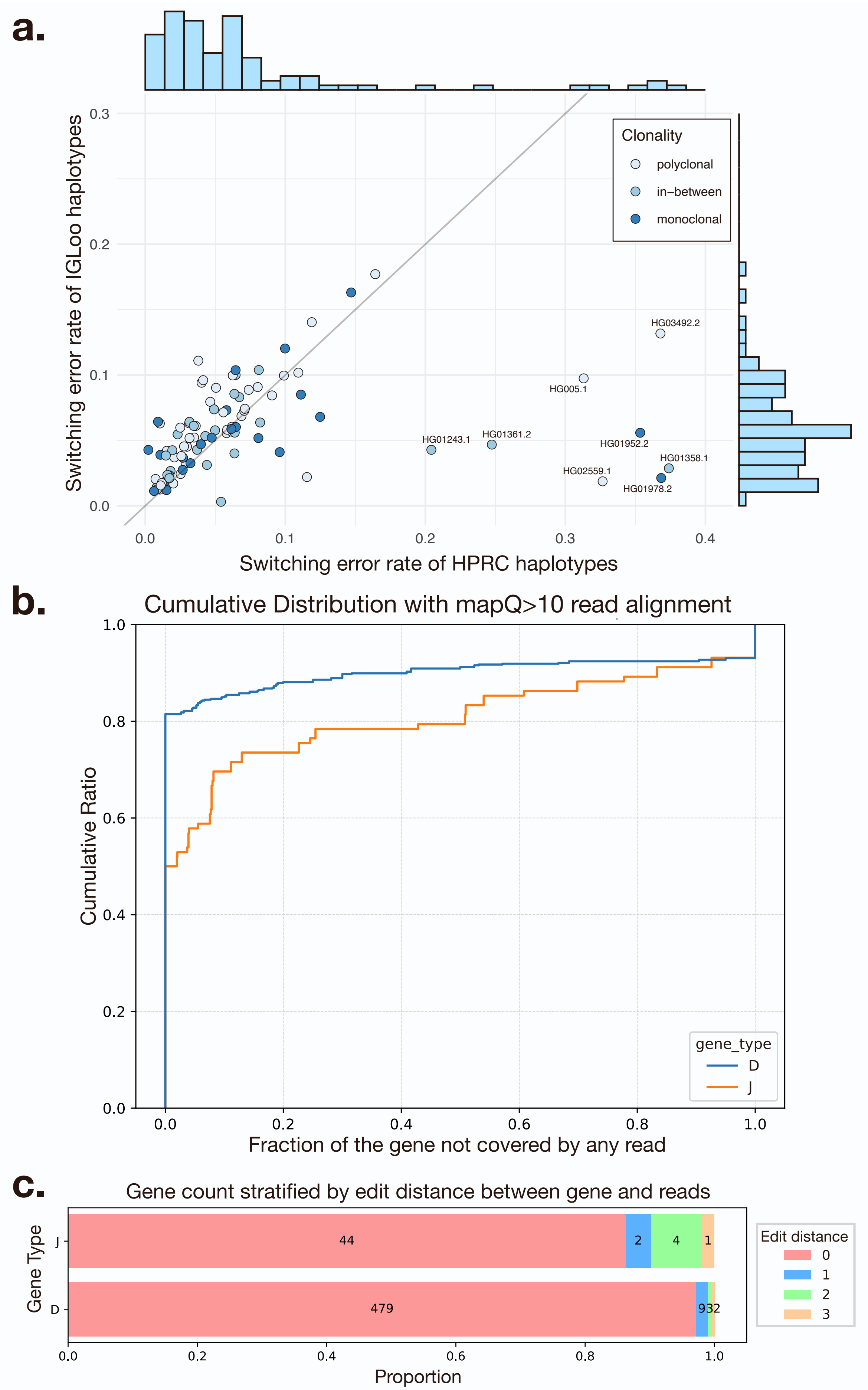
a.
Switching error rate of IGLoo haplotypes
Switching error rate of HPRC haplotypes
0.0
0.1
0.2
0.3
0.4
Clonality
polyclonal
in−between
monoclonal
HG03492.2
HG005.1
HG01243.1
HG01361.2
HG01952.2
HG01358.1
HG02559.1
HG01978.2
b.
Cumulative Distribution with mapQ>10 read alignment
Cumulative Ratio
Fraction of the gene not covered by any read
0.0
0.2
0.4
0.6
0.8
1.0
gene_type
D
J
c.
Gene count stratified by edit distance between gene and reads
Gene Type
J
D
44
2
4
1
479
932
Proportion
0.0
0.2
0.4
0.6
0.8
1.0
Edit distance
0
1
2
3

Figure S12. Evaluation of IGLoo assemblies

a. The switching error rate between HPRC assemblies and IGLoo reassemblies in the IGH region. The hue of the samples represents their clonality, as defined in the Result section: Clonality of the cell lines. **b-c**: Short read evaluation on the newly assembled IGH gene segments. **b**. ratio of the D and J genes being covered by short read realignment. **c**. the edit distance between short reads and the assemblies for the fully covered D and J genes.

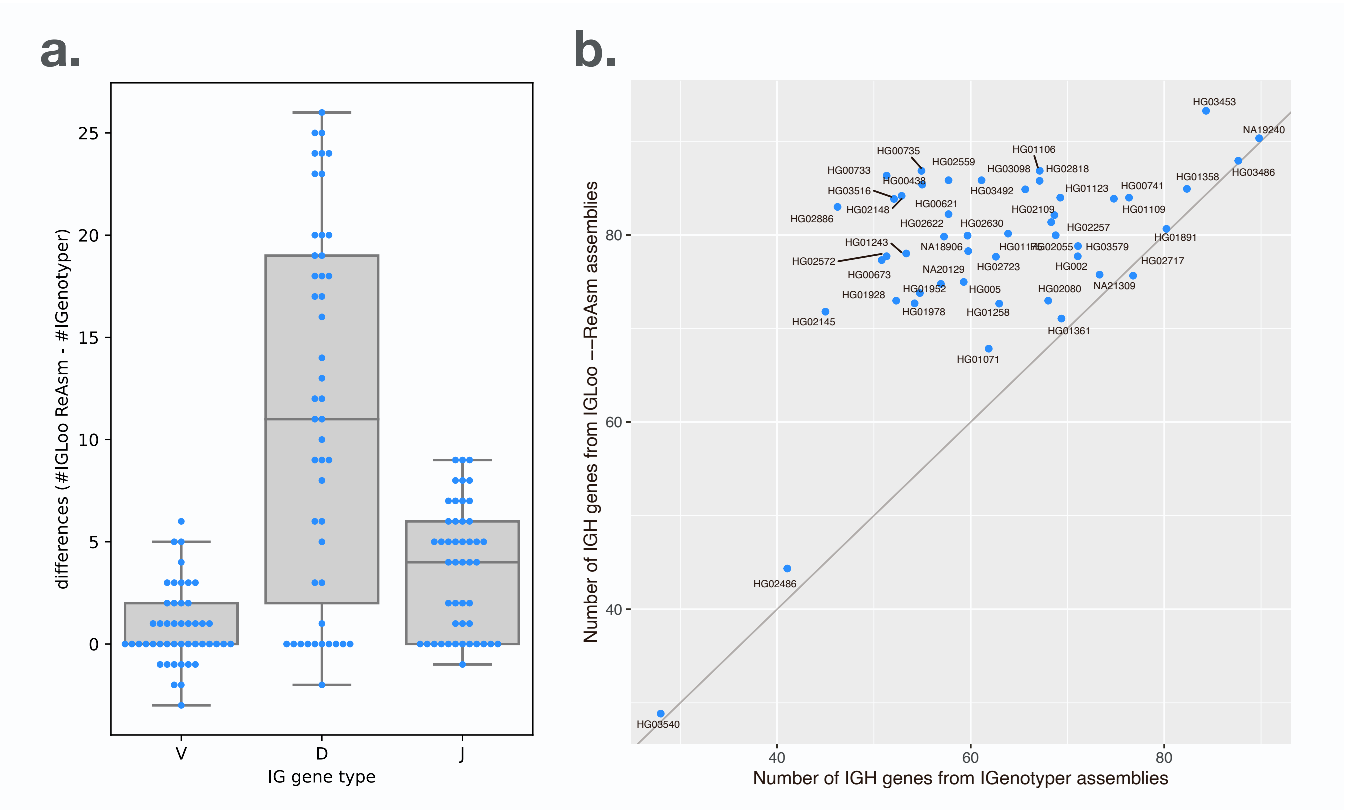

Figure S13. Comparison of IGLoo and IGenotyper

The IGH gene number comparison between IGenotyper[14] and `IGLoo --ReAsm` in the 47 HPRC samples. **a.** the V, D, and J gene differences between two methods in IGH locus. The positive value means `IGLoo --ReAsm` assemblies cover more IGH gene segments while the negative value means IGenotyper assemblies cover more genes. **b.** the total number of IGH V, D, and J genes comparison of the two methods.

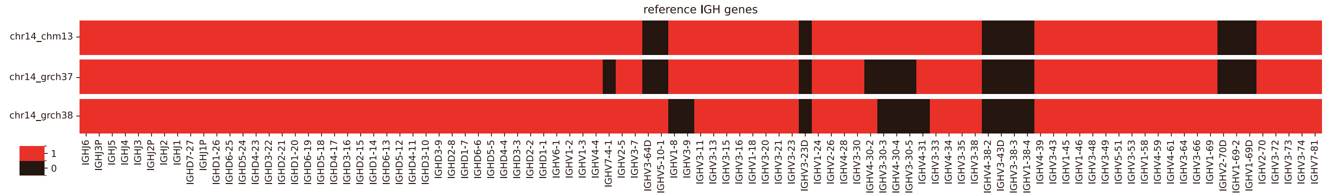

Figure S14. Genotype of the reference genome T2T-CHM13, GRCh37, and GRCh38

the gene number in its relative position on the chromosome 14 of reference genomes T2T-CHM13, GRCh37, and GRCh38. All D and J genes are listed, but only functional or ORF V genes are listed for simplicity
